# DiST: disentangling structure via self-supervised tensors for heterophilous spatial transcriptomics

**DOI:** 10.1093/bib/bbag600

**Published:** 2026-10-30

**Authors:** Yaozhang Liu, Yu Hu, Jinyu Liu, Shuaishuai Tan, Xiaoping Li, Guan Huang, Mai Chen, Langsheng Lei, Jun Du

**Affiliations:** School of Computer Science, Guangdong University of Technology, No. 100 Waihuan Xi Road, Guangzhou Higher Education Mega Center, Panyu District, 510006, Guangdong, China; School of Computer Science, Guangdong University of Technology, No. 100 Waihuan Xi Road, Guangzhou Higher Education Mega Center, Panyu District, 510006, Guangdong, China; School of Computer Science, Guangdong University of Technology, No. 100 Waihuan Xi Road, Guangzhou Higher Education Mega Center, Panyu District, 510006, Guangdong, China; School of Computer Science, Guangdong University of Technology, No. 100 Waihuan Xi Road, Guangzhou Higher Education Mega Center, Panyu District, 510006, Guangdong, China; School of Computer Science, Guangdong University of Technology, No. 100 Waihuan Xi Road, Guangzhou Higher Education Mega Center, Panyu District, 510006, Guangdong, China; Shenzhen TENCLASS Technology Co., Ltd., 8th Floor, Building 5D, Shenzhen Software Base, No. 11, 13, 15 Haitian 1st Road, Yuehai Subdistrict, Nanshan District, 518000, Guangdong, China; Shenzhen TENCLASS Technology Co., Ltd., 8th Floor, Building 5D, Shenzhen Software Base, No. 11, 13, 15 Haitian 1st Road, Yuehai Subdistrict, Nanshan District, 518000, Guangdong, China; Shenzhen TENCLASS Technology Co., Ltd., 8th Floor, Building 5D, Shenzhen Software Base, No. 11, 13, 15 Haitian 1st Road, Yuehai Subdistrict, Nanshan District, 518000, Guangdong, China; Shenzhen TENCLASS Technology Co., Ltd., 8th Floor, Building 5D, Shenzhen Software Base, No. 11, 13, 15 Haitian 1st Road, Yuehai Subdistrict, Nanshan District, 518000, Guangdong, China

**Keywords:** spatially resolved transcriptomics, clustering, graph learning, tensor decompositions, self-supervised learning

## Abstract

Spatially resolved transcriptomics (SRT) serves as a transformative technology for examining gene expression within tissue architecture. However, accurate spatial domain identification remains challenging due to the inherent data sparsity and the tendency of graph-based methods to over-smooth representations at tissue boundaries. To overcome these limitations, we propose DiST, a novel self-supervised tensor learning framework. Distinct from conventional approaches that rely on neighborhood propagation, DiST introduces a Semantically Aligned Ego-Network Tensor construction strategy. By stacking semantically similar ego-networks from the global context, we transform flat graph data into high-order tensor structures that preserve local heterogeneity. Furthermore, we employ a robust low-rank and sparse decomposition mechanism to separate a coherent component used for representation learning from sparse entrywise deviations associated with sequencing dropouts and technical artifacts. This decomposition yields sparsity-resilient representations that recover sharp tissue structures. Extensive experiments on five benchmark datasets demonstrate that DiST outperforms representative methods in spatial clustering. Moreover, downstream biological validation on human breast cancer data confirms DiST’s capability to uncover biologically meaningful molecular signatures and functional heterogeneity.

## Introduction

Recently, spatially resolved transcriptomics (SRT) has transformed bioinformatics by capturing the spatial context of gene expression within tissues [1]. Unlike traditional bulk or single-cell sequencing, SRT maps gene activity to physical locations, enabling researchers to uncover complex tissue architectures and cell–cell interactions [2]. Despite these capabilities, analyzing SRT data remains a significant challenge. The data are characterized by high dimensionality and inherent noise, often suffering from low capture efficiency known as dropouts [3].

To decipher these complex data, graph-based representation learning has become the dominant approach. Methods such as SEDR [4] and SpaGCN [5] construct spatial neighbor graphs and utilize graph neural networks (GNNs) [6] to aggregate gene expression features from neighboring spots. By smoothing expression profiles over spatial neighborhoods, these methods effectively reduce noise in homogeneous tissue regions [7]. However, this smoothing strategy becomes a double-edged sword, especially at the boundaries of different spatial domains [1].

A critical challenge faced by existing GNN-based methods stems from their reliance on the assumption of spatial homophily, where connected nodes are expected to share similar labels or features [8]. While this assumption holds within a homogeneous tissue domain, it breaks down at boundaries where cells interact with neighbors from distinct functional regions. As quantifiably shown in Fig. 1, the heterophily rate of boundary spots and the inter-domain edge ratio increase markedly as the number of spatial neighbors grows, whereas intra-domain spots remain strongly homophilous. To define these regional measures explicitly, let $y_{i} \in \{1, \dots , C\}$ denote the ground-truth domain label of spot *i* and $\mathcal{N}(i)$ its set of spatial neighbors in the *k*-nearest-neighbor spatial graph. A spot is called a *boundary spot* if at least one of its spatial neighbors belongs to a different domain, and an *intra-domain spot* otherwise; let $\mathcal{B}^{in}$ and $\mathcal{B}^{bd}$ denote the sets of intra-domain and boundary spots, respectively. The *heterophily rate* of spot *i* is the fraction of its neighbors from a different domain, $h_{i} = |\{j \in \mathcal{N}(i): y_{j} \neq y_{i}\}| / |\mathcal{N}(i)|$. Figure 1 reports the average heterophily rate of intra-domain spots, $\bar{h}_{\mathcal{B}^{in}} = \frac{1}{|\mathcal{B}^{in}|} \sum _{i \in \mathcal{B}^{in}} h_{i}$, the average heterophily rate of boundary spots, $\bar{h}_{\mathcal{B}^{bd}} = \frac{1}{|\mathcal{B}^{bd}|} \sum _{i \in \mathcal{B}^{bd}} h_{i}$, and the *inter-domain edge ratio*, i.e. the fraction of edges in the *k*-nearest-neighbor graph that connect spots from different domains, $h_{\textrm{edge}} = |\{(u,v) \in \mathcal{E}: y_{u} \neq y_{v}\}| / |\mathcal{E}|$. Since the *k*-nearest-neighbor spatial graph is constructed exclusively from spatial coordinates, its topology—and therefore the label-based heterophily measured on it—does not depend on the expression sparsity of a particular sequencing platform. The observed boundary heterophily is thus a structural property of tissue organization, whereas platform-specific sparsity affects the reliability of observed node features rather than the graph’s heterophily. This indicates that expanding the receptive field to capture broader context introduces a substantial proportion of cross-domain neighbors whose signals can be detrimental when aggregated indiscriminately. However, standard message passing mechanisms generally lack the capability to filter these heterophilous signals, leading to the indiscriminate aggregation of diverse features [9]. Consequently, the representations of boundary spots become mixtures of different domains, resulting in over-smoothed and blurred boundaries [10].

**Figure 1:**
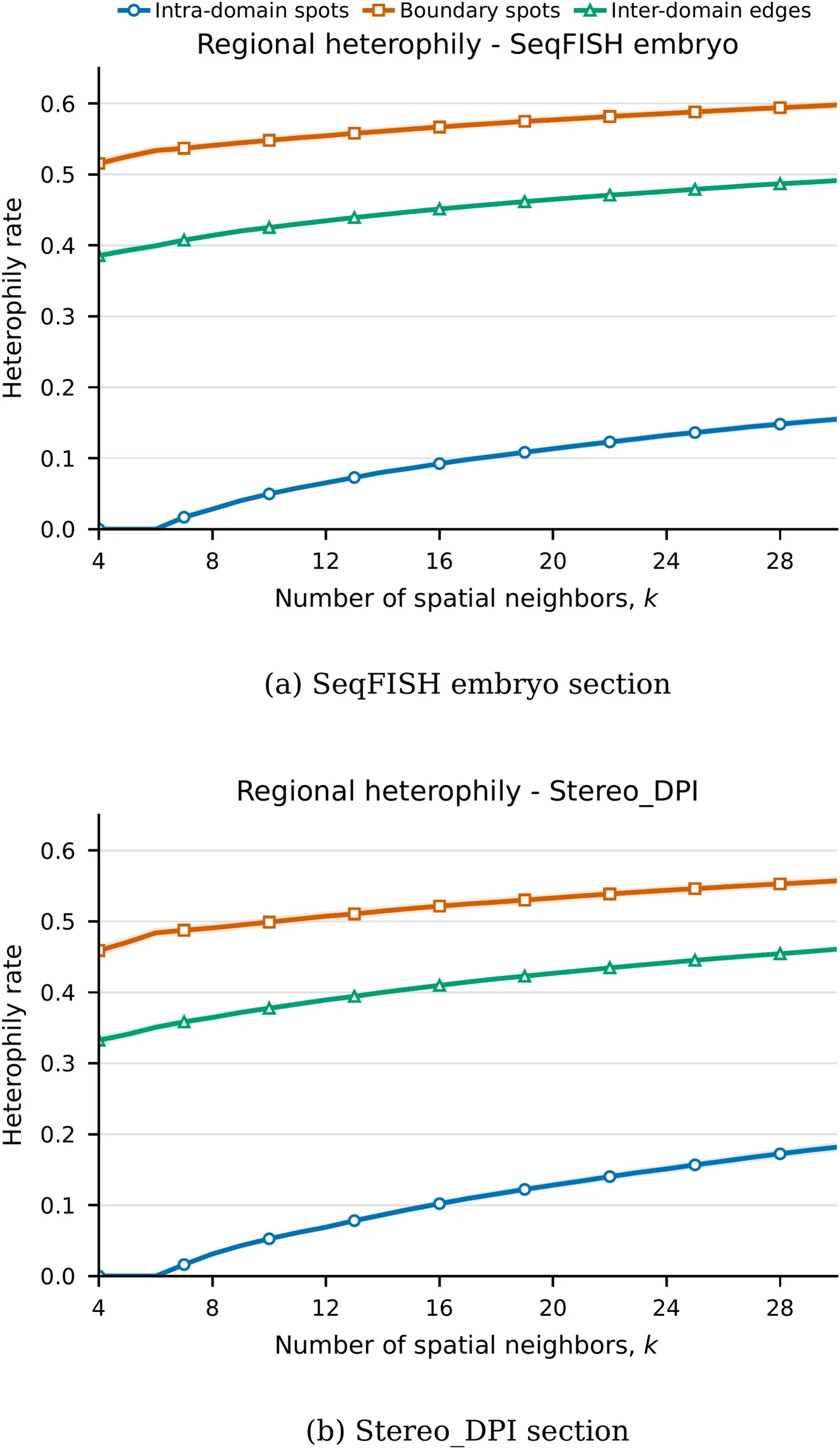
Regional heterophily analysis. Heterophily rate as a function of the number of spatial neighbors (*k*) for three regional components: intra-domain spots, boundary spots, and inter-domain edges. Results are shown for the (a) SeqFISH embryo and (b) Stereo_DPI datasets. Boundary spots and inter-domain edges exhibit substantially higher heterophily than intra-domain spots, and all three measures increase with *k*, quantitatively confirming that expanding the receptive field inevitably mixes signals from distinct spatial domains. Formal definitions of the three measures are provided in the text.

This challenge is further compounded by the severe sparsity of SRT data. Because the spatial graph is constructed from coordinates, its topology is not altered by expression dropouts; however, the observed features used for message passing and semantic similarity estimation become less reliable. When GNNs [6] propagate these incomplete features, they risk amplifying technical variation rather than enhancing the biological signal. Conventional attention mechanisms also struggle here, as sparse and noisy features may yield unreliable attention weights. Therefore, a method that can preserve local heterogeneity while being robust to expression sparsity is urgently needed.

To this end, we propose DiST, a framework designed to learn distinct spot representations by modeling the high-order structure of local neighborhoods. Instead of relying only on conventional neighborhood aggregation [6], DiST constructs a multi-view tensor for each target spot from semantically retrieved spatial ego-networks and three propagation levels. A low-rank and sparse tensor model then regularizes the correlated component while assigning entrywise deviations to a sparse component. This design provides a structured representation that remains robust in the presence of expression sparsity without asserting perfect signal-noise separation.

In summary, our contributions are four-fold:

- We identify that the homophily assumption in existing GNN-based SRT methods leads to over-smoothing at tissue boundaries, a problem validated by our heterophily analysis.
- We propose DiST, a self-supervised framework that utilizes tensor modeling of local ego-networks to capture high-order spatial contexts without blurring local details.
- We introduce a low-rank and sparse tensor decomposition mechanism that regularizes correlated cross-view structure and sparse entrywise deviations, improving resilience to sequencing dropouts.
- We conduct extensive experiments on various SRT datasets. The results demonstrate that DiST significantly outperforms baselines in identifying spatial domains and preserving sharp tissue boundaries.

## Related work

### Spatial domain identification with graph neural networks

Early computational approaches for spatial domain identification primarily relied on probabilistic graphical models [11]. While theoretically grounded, these methods often incur high computational costs and scale poorly. Recently, GNNs have become the dominant paradigm due to their ability to model nonlinear cell–cell interactions [5]. State-of-the-art methods, such as SpaGCN [5] and STAGATE [7], typically construct adjacency graphs based on spatial proximity and employ recursive message-passing mechanisms to aggregate gene expression features.

Recent advancements continue to refine these spatial graphs to capture multi-scale structural dependencies. For instance, SpaNCMG [12] constructs a neighborhood-complementary mixed-view graph by integrating local KNN topologies with global radius-based structures via an attention mechanism.

While effective in homogeneous tissues, these methods face a fundamental limitation rooted in the *homophily assumption* [13]. As indicated in our analysis (Fig. 1), SRT data exhibit high heterophily, particularly at tissue boundaries where neighbors often belong to distinct cell types. Standard GNNs indiscriminately propagate signals across these boundaries, leading to the over-smoothing problem [9]. Although GNN architectures designed for heterophilous graphs have been proposed in the general machine learning community [8, 14, 15], their adaptation to the specific biological context of SRT remains limited. Instead of forcing aggregation, our work seeks a representation learning paradigm that respects local heterogeneity.

### Tensor-based high-order structural modeling

Beyond deep learning frameworks, matrix factorization has been widely explored to uncover latent structures and cluster assignments in ST data. For instance, recent methods like ST-SCSR [16] utilize matrix tri-factorization to simultaneously decompose gene expression profiles and spatial networks, learning an affinity graph through sparse self-representation. While these matrix-based approaches successfully fuse multi-modal features, they are fundamentally constrained to pairwise interactions and inherently flatten complex spatial neighborhoods into 2D representations.

To overcome the limitations of matrix flattening, tensor algebra offers a powerful framework for modeling high-order interactions and multi-dimensional structures without loss of topological integrity [17]. In bioinformatics, tensors have been successfully applied to multi-omics integration and gene regulatory network inference [18, 19].

However, the application of tensor modeling to represent the local ego-networks [20] in SRT is a novel direction. A key advantage of tensor modeling over matrix factorization or graph propagation is its ability to capture complex, multi-view correlations without enforcing smoothness. By modeling the local neighborhood as a high-order tensor, we can leverage tensor decomposition techniques (e.g. Tucker or CP decomposition) to extract latent structural factors [17]. This process effectively disentangles the underlying biological patterns from the complex spatial context, offering a mathematically rigorous alternative to the heuristic smoothing operations used in standard GNNs and the dimensional collapse seen in matrix methods.

### Self-supervised learning on graphs

Self-supervised learning (SSL) on graphs is typically categorized into contrastive and generative approaches, both of which have been explored to decipher spatial transcriptomics.

Contrastive methods aim to learn robust embeddings by increasing agreement between local and global representations. For instance, SCGDL [21] adapts the Deep Graph Infomax framework by employing a residual gated graph convolutional network, subsequently applying a Bayesian Gaussian mixture model to identify spatial domains. Similarly, ST-GCP [22] proposes a joint representation learning framework that relies on a structure-feature perturbation mechanism, specifically employing random edge dropouts and feature permutations to create and align augmented graph views. However, these conventional contrastive frameworks heavily rely on such heuristic data augmentations. In heterophilous biological networks, randomly dropping edges or masking features risks irrevocably destroying critical semantic boundaries and localized topological structures [23].

On the other hand, generative methods (e.g. GAE [24], GraphMAE [25]) focus on reconstructing input data to learn latent representations. When adapting these frameworks to spatial transcriptomics, forcing the reconstruction of raw, noisy spatial data may inadvertently overfit the model to technical artifacts, such as prevalent dropouts and sequencing noise.

**Distinct from these paradigms, our proposed DiST introduces a decomposition-based self-supervised tensor recovery framework.** Instead of maximizing mutual information or reconstructing noisy inputs, DiST employs a *low-rank tensor approximation* objective. This approach uses the low-complexity structure commonly observed in gene-expression data as a modeling prior [26]. By regularizing correlated structure and sparse deviations rather than directly reconstructing every noisy entry, DiST learns robust embeddings suitable for complex spatial domains.

## Problem statement

### Notations

We denote the observed gene expression matrix as $\mathbf{X} \in \mathbb{R}^{N \times M}$, with *N* representing the number of spots and *M* the number of genes. The spatial coordinates for these spots are given by $\mathbf{S} \in \mathbb{R}^{N \times 2}$. Based on the spatial proximity defined in $\mathbf{S}$, we construct a spatial neighborhood graph $\mathcal{G} = (\mathcal{V}, \mathcal{E})$, where $\mathcal{V}$ denotes the set of spots and $\mathcal{E}$ comprises the edges linking adjacent spots. The primary objective of SRT analysis is to derive a latent representation $\mathbf{H} \in \mathbb{R}^{N \times d}$, where *d* is the embedding size, and a spatial-domain assignment vector $\mathbf{y}=(y_{1},\ldots ,y_{N})\in \{1,\ldots ,C\}^{N}$, where *C* is the number of distinct clusters.

### The challenge of heterophily and over-smoothing

The prevailing GNN-based paradigms generally rely on the *homophily assumption*. These methods employ message-passing mechanisms to aggregate information from local neighborhoods, typically formalized as 


(1) \begin{align*}& \begin{aligned} \mathbf{h}_{i}^{(l+1)}=\sigma \left( \sum_{j\in \mathcal{N} (i)\cup \{i\}}{\frac{1}{c_{ij}}\mathbf{W}^{(l)}\mathbf{h}_{j}^{(l)}} \right), \end{aligned}\end{align*}

where $\mathcal{N}(i)$ denotes the set of neighbors of spot *i*, $c_{ij}$ is a normalization constant, and $\sigma (\cdot )$ is an activation function. Implicitly, this aggregation process minimizes the feature divergence between connected nodes. Nevertheless, our empirical observations indicate that SRT graphs display significant heterophily, especially near tissue boundaries [10, 27]. In such transition zones, a spot *i* and its neighbor *j* frequently originate from different spatial domains (i.e. $y_{i} \neq y_{j}$). In these scenarios, applying standard aggregation indiscriminately blends signals from divergent distributions. Consequently, the learned embeddings suffer from *over-smoothing* [28, 29], causing $\mathbf{H}$ to lose local discriminative capacity and blurring the sharp boundaries between domains.

### Problem formulation

Given the zero-inflated expression matrix $\mathbf{X}$ and the spatial graph $\mathcal{G}$, our goal is to jointly address graph heterophily and expression sparsity. We use the tensor-based self-supervised framework DiST to learn a mapping $f:(\mathbf{X},\mathcal{G})\mapsto \mathbf{H}$. The representation $\mathbf{H}$ is designed to satisfy two criteria: (i) **structure recovery**: it should retain correlated expression structure while reducing the influence of sparse technical artifacts; (ii) **boundary preservation**: it must retain local heterogeneity at tissue boundaries, preventing the loss of detail caused by indiscriminate global graph aggregation.

## Method

As illustrated in Fig. 2, DiST is a tensor-based framework for disentangling coherent biological signals from heterophilous and noisy spatial transcriptomics data. The framework consists of three stages. First, DiST constructs heterophilous local ego-networks for each sample and organizes transcriptomically similar, potentially spatially distant ego-networks into a semantically aligned tensor representation. Second, the ego-network tensor is decomposed via SSL into a low-rank component used for representation extraction and a sparse component modeling entrywise deviations, without relying on an additional graph-smoothing layer. Finally, the resulting low-rank representations are used to derive sample embeddings for downstream analyses such as spatial domain identification and differential gene expression analysis.

**Figure 2:**
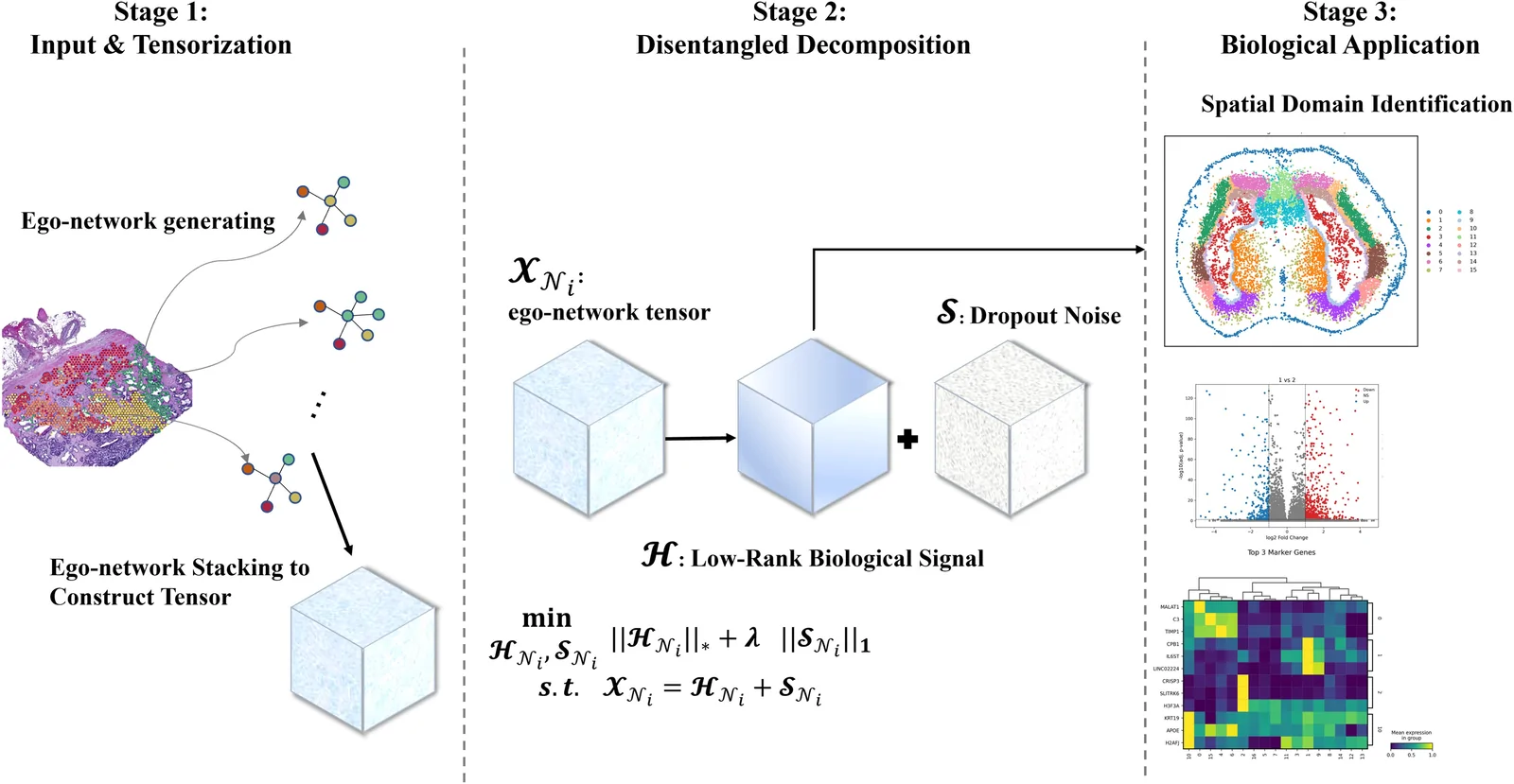
Given spatial transcriptomics data, a heterophilous local ego-network is constructed for each sample to capture diverse and locally heterogeneous neighborhood contexts. The resulting ego-networks are then organized into a semantically aligned ego-network tensor $\boldsymbol{\mathcal{X}}_{i}$. Stage 2 (Disentangled Decomposition): the ego-network tensor is decomposed via self-supervised optimization into a low-rank tensor $\boldsymbol{\mathcal{H}}_{i}$, used for representation extraction, and a sparse tensor $\boldsymbol{\mathcal{S}}_{i}$, used to model entrywise deviations such as dropout-associated artifacts. Stage 3 (Biological Application): regularized low-rank representations are used to derive embeddings of central samples, enabling downstream biological analyses such as spatial domain identification and differential gene expression analysis.

### Spatial ego-network and local propagation

To address the over-smoothing issue inherent in global scalar propagation, we first shift the focus from the entire graph to local spatial ego-networks. In standard global propagation, signals from distinct spatial domains are inevitably mixed across heterophilous boundaries. Ideally, the propagation structure should be block-diagonal to strictly restrict intra-class aggregation. However, explicitly constructing such a perfect graph structure from highly noisy data is mathematically ill-posed.

To bypass this arduous task, we localize the problem to explicitly optimize the representation matrix of local niches.

**Spatial ego-network.** For each spot *i*, we define its spatial ego-network $\mathcal{G}^{sp}_{i}$ as the local patch induced by *i* and its 1-hop spatial neighbors $\mathcal{N}(i)$. We represent its nodes by an ordered list $\mathcal{N}^{sp}_{i}=(i,n_{i,2},\ldots )$, in which the central spot is moved to the first position and the remaining nodes retain the deterministic order returned by the subgraph extraction routine. For a target spot *i* and its semantic-anchor set $\Omega _{i}$, the implementation uses the target-specific padded size $D_{i}=1+\max _{j\in \Omega _{i}}\deg _{\mathcal{G}_{\!+}}(j)$, where $\mathcal{G}_{\!+}$ is the NetworkX graph constructed from the self-looped adjacency matrix and $\deg _{\mathcal{G}_{\!+}}$ is the degree returned by NetworkX (including the self-loop contribution under its degree convention). Each ego-network associated with $\Omega _{i}$ is truncated or zero-padded to $D_{i}$ rows. Thus, $\mathbf{X}_{\mathcal{N}^{sp}_{j}}\in \mathbb{R}^{D_{i}\times M}$ denotes the padded feature matrix of anchor $j\in \Omega _{i}$. Zero padding standardizes the array size but is not interpreted as an additional graph node.

**Low-rank property of local propagation.** Within a spatial ego-network, a generic local propagation can be written as $\mathbf{H}_{\mathcal{N}^{sp}_{i}}=\mathbf{P}_{\mathcal{N}^{sp}_{i}}\mathbf{X}_{\mathcal{N}^{sp}_{i}}$, where $\mathbf{P}_{\mathcal{N}^{sp}_{i}}\in \mathbb{R}^{D_{i}\times D_{i}}$ is a patch-level propagation operator. Repeated graph propagation is known to emphasize a restricted spectral subspace [20], which motivates the use of a low-complexity prior. This observation is motivational rather than a proof that every propagated patch is exactly low-rank. In the implementation below, the multi-hop features are generated by the unnormalized global adjacency matrix, and low complexity is imposed explicitly by the tensor decomposition.

### Spatial ego-network and semantic tensor construction

An insight of DiST is that relying solely on a single local spatial patch limits the model’s ability to distinguish robust biological signals from artifacts, as functionally similar tissues are often spatially disjoint. Furthermore, traditional global scalar propagation forces assimilation with heterophilous neighbors, leading to severe over-smoothing. To capture comprehensive structural dependencies without compromising domain boundaries, we propose a multi-view tensor construction mechanism that aligns local spatial ego-networks across global semantic similarities and multiple receptive fields.

**Global semantic anchor retrieval.** We calculate pairwise cosine similarity using the input highly variable-gene feature vectors. For each target spot *i*, the implementation retrieves the top *K=12* spots over the full dataset, explicitly inserts *i* if necessary, and moves it to the first position. We denote the resulting ordered anchor list by $\Omega _{i}=(i,j_{1},\ldots ,j_{K-1})$. The retrieval is based on transcriptomic similarity and does not require the anchors to be spatially adjacent.

**Multi-hop context and spatial ego-network extraction.** For each anchor $j \in \Omega _{i}$, we extract its 1-hop spatial ego-network $\mathcal{G}^{sp}_{j}$ and standardize it to $D_{i}$ rows as defined above. We then compute three feature levels on the global graph: $\mathbf{X}^{(0)}=\mathbf{X}$, $\mathbf{X}^{(1)}=\mathbf{A}\mathbf{X}$, and $\mathbf{X}^{(2)}=\mathbf{A}^{2}\mathbf{X}$, where $\mathbf{A}$ is the binary spatial adjacency matrix after adding self-loops. Consistent with the released implementation, no degree normalization is applied in these two propagation steps. For each ordered ego-network $\mathcal{N}^{sp}_{j}$, we extract $\mathbf{X}^{(h)}_{\mathcal{N}^{sp}_{j}}\in \mathbb{R}^{D_{i}\times M}$ for $h\in \{0,1,2\}$. These propagated features may mix cross-domain signals; the decomposition below is used as a regularized separation rather than as a guarantee that every heterophilous entry is removed.

**Multi-view tensor stacking.** We construct a third-order tensor $\boldsymbol{\mathcal{X}}_{i}$ by stacking the matrices along the first mode in the exact implementation order: all *K* anchors at level *h=0*, followed by all anchors at *h=1*, and then all anchors at *h=2*. Hence, $\boldsymbol{\mathcal{X}}_{i}\in \mathbb{R}^{3K\times D_{i}\times M}$ and 


(2) \begin{align*}& \boldsymbol{\mathcal{X}}_{i} = \operatorname{Stack}_{1}\!\left(\left(\mathbf{X}^{(h)}_{\mathcal{N}^{sp}_{j}}\right)_{h=0,1,2;\,j\in\Omega_{i}}\right).\end{align*}

**Motivation and scope of the tensor regularizer.** The first mode indexes the *K* semantic anchors at three propagation levels, the second mode indexes the $D_{i}$ ordered and padded positions within each ego-network, and the third mode indexes the *M* genes. Keeping these axes separate avoids flattening node and gene coordinates before regularization. The construction does not, however, imply a one-to-one biological correspondence between noncentral neighbor positions across different ego-networks; only the central position and the view ordering are explicitly aligned.

The GTRPCA backend applies a t-product-based tensor nuclear norm (TNN) to this fixed array layout [30]. Under the implemented shape $3K\times D_{i}\times M$, the transform used by the TNN is taken along the third mode. We use this operation as a convex low-complexity regularizer and do not assign a biological interpretation to individual Fourier frequencies of the gene index. Let $\mathbf{H}_{i,\textrm{mat}}\in \mathbb{R}^{3K\times D_{i}M}$ denote the matrix obtained by flattening the last two modes. Matrix robust principal component analysis (PCA) imposes one matrix nuclear norm on this flattened representation, whereas DiST retains the three-way layout and applies the TNN used by the tensor solver. Neither regularizer universally dominates the other for arbitrary tensors; the practical benefit of retaining the tensor layout is therefore established by the controlled Matrix-DiST ablation rather than by a universal rank or norm inequality.

Within this model, the low-rank term favors structure shared across the stacked views, while the entrywise $\ell _{1}$ term absorbs sparse deviations. This is a modeling assumption, not a claim that every dropout or heterophilous signal is exactly sparse or perfectly separable. The downstream biological and ablation analyses provide empirical evidence for the usefulness of the recovered representation.

### Sparse-constrained tensor structure learning

The constructed tensor $\boldsymbol{\mathcal{X}}_{i}$ combines correlated expression structure with entrywise expression sparsity and other deviations. Consistent with our problem formulation, we model it as a superposition of a low-rank component and a sparse component: 


(3) \begin{align*}& \begin{aligned} \boldsymbol{\mathcal{X}}_{i} = \boldsymbol{\mathcal{H}}_{i} + \boldsymbol{\mathcal{S}}_{i}, \end{aligned}\end{align*}

where:

-
$\boldsymbol{\mathcal{H}}_{i} \in \mathbb{R}^{3K \times D_{i} \times M}$ is the **low-rank tensor**, which provides the regularized component used for representation extraction.
-
$\boldsymbol{\mathcal{S}}_{i} \in \mathbb{R}^{3K \times D_{i} \times M}$ is the **sparse artifact tensor**, which models sparse entrywise deviations from the low-rank component, including dropout-associated and technical artifacts.

**Optimization objective.** To recover $\boldsymbol{\mathcal{H}}_{i}$, we minimize a joint objective function: 


(4) \begin{align*}& \begin{aligned} &\min_{\boldsymbol{\mathcal{H}}_{i}, \boldsymbol{\mathcal{S}}_{i}} \|\boldsymbol{\mathcal{H}}_{i}\|_{*} + \lambda_{i} \|\boldsymbol{\mathcal{S}}_{i}\|_{1} \\ &\mathrm{s.t.} \quad \boldsymbol{\mathcal{X}}_{i} = \boldsymbol{\mathcal{H}}_{i} + \boldsymbol{\mathcal{S}}_{i}, \end{aligned}\end{align*}

where $\|\cdot \|_{1}$ is the $\ell _{1}$-norm promoting an entrywise sparse component and $\|\cdot \|_{*}$ denotes the TNN based on the t-product definition [30].

Mathematically, $\|\boldsymbol{\mathcal{H}}_{i}\|_{*}$ denotes the TNN convention implemented by the GTRPCA solver. It can be evaluated through singular-value thresholding of the transformed frontal slices associated with the block-circulant representation. In the implemented tensor layout, semantic anchors and propagation levels are stacked in the first mode, not the third mode. Accordingly, we interpret the TNN as a joint low-complexity regularizer on the complete three-way array, without claiming that the transformed gene frequencies themselves have a direct biological meaning.

**Self-supervised interpretation.** We clarify why DiST is categorized as self-supervised rather than merely unsupervised or reconstruction-based. Following the broad definition of SSL, the supervisory signals used by DiST are generated entirely from the observed data itself, without any external spatial-domain or cell-type annotations, and DiST exploits two forms of such data-derived supervision. First, for every target spot *i*, the ordered semantic-anchor list $\Omega _{i}=(i,j_{1},\ldots ,j_{K-1})$ is constructed automatically from transcriptomic similarity. These data-derived semantic correspondences provide relational supervision among potentially distant ego-networks without requiring labels. Second, DiST learns spot representations through a proxy tensor recovery task, i.e. the objective in (4), in which the observed tensor $\boldsymbol{\mathcal{X}}_{i}$ supplies the observation-consistency signal, while the low-rank and sparse priors regularize cross-view structure and entrywise deviations. Importantly, this recovery objective does not use or optimize the downstream spatial-domain labels, and the recovered tensor is subsequently used only to derive representations for clustering. DiST can therefore be viewed as a decomposition-based, non-contrastive self-supervised framework: it is unsupervised with respect to the downstream task, yet its representation-learning objective is self-supervised, because both the semantic relations and the recovery constraints are derived from the data itself.

### Optimization via alternating direction method of multipliers

The optimization problem above is convex. We employ the alternating direction method of multipliers (ADMM) to solve it efficiently. The augmented Lagrangian function is defined as 


(5) \begin{align*}& \begin{aligned} \mathcal{L}(\boldsymbol{\mathcal{H}}_{i}, \boldsymbol{\mathcal{S}}_{i}, \boldsymbol{\Lambda}_{i}, \rho) = \ & \|\boldsymbol{\mathcal{H}}_{i}\|_{*} + \lambda_{i} \|\boldsymbol{\mathcal{S}}_{i}\|_{1} + \langle \boldsymbol{\Lambda}_{i}, \boldsymbol{\mathcal{H}}_{i} + \boldsymbol{\mathcal{S}}_{i} - \boldsymbol{\mathcal{X}}_{i} \rangle \\ & + \frac{\rho}{2}\|\boldsymbol{\mathcal{H}}_{i} + \boldsymbol{\mathcal{S}}_{i} - \boldsymbol{\mathcal{X}}_{i}\|_{F}^{2}, \end{aligned}\end{align*}

where $\boldsymbol{\Lambda }_{i}$ is the Lagrange multiplier tensor associated with target tensor *i* and *ρ> 0* is the penalty parameter. The standard ADMM updates associated with this objective are as follows:

**(1) Updating $ \boldsymbol{\mathcal{H}}_{i}$ (biological structure)**


(6) \begin{align*}& \begin{aligned} \boldsymbol{\mathcal{H}}_{i}^{t+1} = \arg\min_{\boldsymbol{\mathcal{H}}_{i}} \ \frac{1}{2}\|\boldsymbol{\mathcal{H}}_{i} + \boldsymbol{\mathcal{S}}_{i}^{t} - \boldsymbol{\mathcal{X}}_{i} + \boldsymbol{\Lambda}_{i}^{t}/\rho^{t}\|_{F}^{2} + \frac{1}{\rho^{t}}\|\boldsymbol{\mathcal{H}}_{i}\|_{*}. \end{aligned}\end{align*}

This subproblem has a closed-form solution via the tensor singular value thresholding operator defined in [30]: 


(7) \begin{align*}& \boldsymbol{\mathcal{H}}_{i}^{t+1} = \mathcal{D}_{1/\rho^{t}}(\boldsymbol{\mathcal{X}}_{i} - \boldsymbol{\mathcal{S}}_{i}^{t} - \boldsymbol{\Lambda}_{i}^{t}/\rho^{t}).\end{align*}

**(2) Updating $ \boldsymbol{\mathcal{S}}_{i}$ (sparse artifacts)**


(8) \begin{align*}& \begin{aligned} \boldsymbol{\mathcal{S}}_{i}^{t+1} = \arg\min_{\boldsymbol{\mathcal{S}}_{i}} \ \frac{1}{2}\|\boldsymbol{\mathcal{H}}_{i}^{t+1} + \boldsymbol{\mathcal{S}}_{i} - \boldsymbol{\mathcal{X}}_{i} + \boldsymbol{\Lambda}_{i}^{t}/\rho^{t}\|_{F}^{2} + \frac{\lambda_{i}}{\rho^{t}}\|\boldsymbol{\mathcal{S}}_{i}\|_{1}. \end{aligned}\end{align*}

This subproblem is solved element-wise using the Soft-Thresholding operator $\mathcal{T}_{\tau }(\cdot )$ defined in [30], shrinking entrywise deviations toward zero: 


(9) \begin{align*}& \boldsymbol{\mathcal{S}}_{i}^{t+1} = \mathcal{T}_{\lambda_{i}/\rho^{t}}(\boldsymbol{\mathcal{X}}_{i} - \boldsymbol{\mathcal{H}}_{i}^{t+1} - \boldsymbol{\Lambda}_{i}^{t}/\rho^{t}).\end{align*}

**(3) Updating multipliers $ \boldsymbol{\Lambda }_{i}$ and * ρ***


(10) \begin{align*}& \begin{aligned} & \boldsymbol{\Lambda}_{i}^{t+1} = \boldsymbol{\Lambda}_{i}^{t} + \rho^{t}(\boldsymbol{\mathcal{H}}_{i}^{t+1} + \boldsymbol{\mathcal{S}}_{i}^{t+1} - \boldsymbol{\mathcal{X}}_{i}),\\ & \rho^{t+1} = \min\{\alpha\rho^{t}, \rho^{\textrm{max}}\}, \end{aligned}\end{align*}

where *α =1.1* and $\rho ^{\textrm{max}}=10^{10}$, which is a common choice in previous ADMM algorithms [30].

In the core experimental pipeline, the GTRPCA call is executed with a fixed upper bound of $T_{\max }=10$ ADMM iterations for each target tensor. We therefore denote the low-rank tensor returned at this computational budget by $\hat{\boldsymbol{\mathcal{H}}}_{i}$ and do not assume that every target tensor reaches a common numerical tolerance within the production run. Normalized reconstruction residuals and relative iterate changes are examined separately as *post hoc* optimization diagnostics.

### Representation extraction

After optimization, the tensor $\hat{\boldsymbol{\mathcal{H}}}_{i}$ has shape $3K\times D_{i}\times M$. Because $\Omega _{i}$ begins with target spot *i* and the *h=0* block is stacked first, $\hat{\boldsymbol{\mathcal{H}}}_{i}(1,:,:)$ is the recovered 0-hop ego-network associated with *i*. Its first row is the target spot, so the primary central readout is $\hat{\mathbf{h}}_{i}=\hat{\boldsymbol{\mathcal{H}}}_{i}(1,1,:)\in \mathbb{R}^{M}$. Stacking these vectors gives $\hat{\mathbf{H}}=[\hat{\mathbf{h}}_{1};\ldots ;\hat{\mathbf{h}}_{N}]\in \mathbb{R}^{N\times M}$. The implementation additionally exports two auxiliary readouts: an average over the first *K* recovered 0-hop anchor slices and a sum over the first three recovered 0-hop anchor slices. These outputs are retained for implementation-level comparisons; the mathematical definition above refers to the central readout. The exported feature matrix is normalized first across genes for each spot and then across spots for each gene using $\ell _{2}$ normalization. It may subsequently be compressed to *d* dimensions, e.g. by PCA, to obtain $\mathbf{H}\in \mathbb{R}^{N\times d}$ for clustering.

## Experiment

We evaluate DiST on multiple benchmark datasets to validate its performance in spatial domain identification, robustness to structural sparsity, and ability to preserve tissue boundaries. Furthermore, to demonstrate the biological interpretability of the identified domains, we conduct in-depth differential expression gene (DEG) [31] analysis, multisource pathway and STRING protein–protein interaction (PPI) enrichment analysis, and linkage of the clustering results to an independent single-cell reference specifically on the Human Breast Cancer dataset.

### Experimental setup

**Datasets.** We evaluate DiST on five datasets across diverse platforms to demonstrate its broad applicability: (i) **Human Breast Cancer (10X_BC)** [32] (10x Visium) for delineating tumor heterogeneity; (ii) **Mouse Hippocampus (ST_Hip)** [33] (ST) for identifying laminar anatomical layers; (iii) **Axolotl Brain (Stereo_AB & Stereo_DPI)** [34] (Stereo-seq) for distinguishing fine-grained domains in regenerative tissues; and (iv) **Mouse Embryo (SeqFISH_embryo)** [35] (seqFISH) for validating robustness in complex organogenesis architectures.

**Baselines.** To validate the effectiveness of DiST, we compare it against seven representative baselines, including both SRT-specific methods and general-purpose GNNs designed for heterophilic graphs: (i) **SpaGCN** [5], which fuses histology and spatial coordinates with gene expression using a graph convolutional approach; (ii) **SEDR** [4], a deep representation learning framework that couples a deep autoencoder with a GCN to capture latent features; (iii) **STAGATE** [7], which leverages an attention-based mechanism to model adaptive spatial dependencies between spots; (iv) **DeepST** [36], a hybrid model integrating GNNs with denoising autoencoders for enhanced domain recognition; (v) **GraphST** [37], a graph self-supervised contrastive learning method for spatial domain identification; (vi) **Geom-GCN** [14], a geometric aggregation GNN proposed for node classification on heterophilic graphs; and (vii) **BernNet** [15], a GNN that learns arbitrary graph convolution filters via Bernstein approximation and has been shown to be effective on heterophilic graphs. For all baselines, we executed the experiments using their official implementations and default parameter settings to ensure fair comparison.

**Quantitative metrics.** For all five datasets, we employ two complementary metrics to evaluate the clustering performance and spatial boundary identification. First, the **Adjusted Rand Index (ARI)** [11] is utilized to measure the agreement between the identified spatial domains and the available ground truth or expert annotations, providing a standardized evaluation of overall structural identification accuracy. Second, to explicitly assess the quality of boundary detection, we introduce the **Boundary F1 score (BF1)**. BF1 quantifies the alignment between the boundaries of the predicted spatial domains and the ground truth contours, offering a fine-grained evaluation of how well the model preserves domain interfaces without over-smoothing. Formally, we construct binary boundary masks for both the predicted and the ground-truth domain labels: a spot is declared a boundary spot if at least one of its *κ * nearest spatial neighbors (in physical coordinates) belongs to a different domain, and we set *κ = 6* in our implementation. The two masks are then compared spot by spot, yielding boundary precision $P = \textrm{TP}/(\textrm{TP}+\textrm{FP})$ and boundary recall $R = \textrm{TP}/(\textrm{TP}+\textrm{FN})$, where $\textrm{TP}$, $\textrm{FP,}$ and $\textrm{FN}$ denote the numbers of correctly detected, falsely detected, and missed boundary spots, respectively. The Boundary F1 score is their harmonic mean, $F_{1} = 2PR/(P+R)$.

**Biological validation (case study).** To verify the biological interpretability of the identified domains, we conduct a specific case study on the **Human Breast Cancer** dataset. We perform **DEG** [31] analysis using the Wilcoxon rank-sum test (adjusted *P*-value *<.05*), and further characterize the identified domains through Gene Ontology (GO) Biological Process and multi-source pathway enrichment (KEGG 2021 Human, MSigDB Hallmark 2020, and Reactome 2022), STRING PPI network analysis, and mapping to the independent breast-cancer single-cell reference GSE176078 [38] via Tangram. This analysis aims to confirm that the domains identified by DiST correspond to biologically distinct functional regions aligned with known literature.

### Performance on spatial domain identification

We initiate our experimental analysis by evaluating the overall clustering performance of DiST against seven representative baseline methods. Table 1 summarizes the quantitative results (ARI scores) across all five benchmark datasets covering diverse sequencing platforms.

**Table 1:** Comparison of spatial domain identification performance (ARI) across five datasets.

**Method**	**SeqFISH**	**Stereo_AB**	**Stereo_DPI**	**10X_BC**	**ST_Hip**
SpaGCN	*0.4023 ± 0.0156*	*0.4604 ± 0.0079*	*0.3569 ± 0.0120*	*0.5448 ± 0.0280*	*0.5614 ± 0.0201*
STAGATE	*0.2760 ± 0.0047*	*0.5145 ± 0.0224*	*0.3688 ± 0.0129*	*0.4529 ± 0.0156*	*0.4465 ± 0.0343*
DeepST	*0.3738 ± 0.0318*	*0.5086 ± 0.0116*	*0.3751 ± 0.0205*	*0.5575 ± 0.0179*	*0.4790 ± 0.0322*
SEDR	*0.3648 ± 0.0176*	*0.5018 ± 0.0153*	*0.3568 ± 0.0223*	*0.3942 ± 0.0351*	*0.5201 ± 0.0174*
GraphST	*0.3395 ± 0.0147*	*0.4876 ± 0.0112*	*0.3853 ± 0.0127*	*0.5871 ± 0.0180*	*0.5097 ± 0.0324*
Geom-GCN	*0.2679 ± 0.0119*	*0.5028 ± 0.0186*	*0.3722 ± 0.0114*	*0.5086 ± 0.0184*	*0.5487 ± 0.0418*
BernNet	*0.2430 ± 0.0140*	*0.4411 ± 0.0177*	*0.3649 ± 0.0135*	*0.4558 ± 0.0260*	*0.4677 ± 0.0449*
**DiST (Ours)**	$\mathbf{0.4516 \pm 0.0071}$	$\mathbf{0.5900 \pm 0.0069}$	$\mathbf{0.4288 \pm 0.0087}$	$\mathbf{0.5998 \pm 0.0131}$	$\mathbf{0.6186 \pm 0.0123}$

The results are presented as *Mean *± * Standard Deviation*. The best results are highlighted in **bold**. DiST consistently achieves superior performance across distinct anatomical layers and heterogeneous tissue architectures.

**Quantitative results.** As evidenced in Table 1 and 2, DiST consistently outperforms all competing methods across varying tissue types and technologies, achieving the highest ARI and BF1 scores in every case. This demonstrates the model’s robustness to the distinct noise profiles of 10x Visium, ST, Stereo-seq, and SeqFISH platforms.

**Table 2:** Comparison of spatial boundary identification performance (Boundary F1) across five datasets.

**Method**	**SeqFISH**	**Stereo_AB**	**Stereo_DPI**	**10X_BC**	**ST_Hip**
SpaGCN	*0.7130 ± 0.0072*	*0.6100 ± 0.0128*	*0.6481 ± 0.0207*	*0.6132 ± 0.0075*	*0.7456 ± 0.0214*
STAGATE	*0.4619 ± 0.0194*	*0.5136 ± 0.0217*	*0.5868 ± 0.0127*	*0.5259 ± 0.0211*	*0.7204 ± 0.0193*
DeepST	*0.5866 ± 0.0422*	*0.5672 ± 0.0343*	*0.5766 ± 0.0238*	*0.5802 ± 0.0211*	*0.6455 ± 0.0221*
SEDR	*0.7060 ± 0.0137*	*0.5565 ± 0.0251*	*0.6628 ± 0.0224*	*0.5695 ± 0.0119*	*0.8042 ± 0.0208*
GraphST	*0.6635 ± 0.0142*	*0.6672 ± 0.0146*	*0.6982 ± 0.0138*	*0.5078 ± 0.0155*	*0.7759 ± 0.0233*
Geom-GCN	*0.4674 ± 0.0165*	*0.5599 ± 0.0281*	*0.6406 ± 0.0125*	*0.5571 ± 0.0125*	*0.7905 ± 0.0336*
BernNet	*0.3428 ± 0.0109*	*0.4309 ± 0.0135*	*0.5393 ± 0.0133*	*0.5095 ± 0.0143*	*0.7236 ± 0.0094*
**DiST (Ours)**	$\mathbf{0.7988 \pm 0.0055}$	$\mathbf{0.7287 \pm 0.0061}$	$\mathbf{0.8214 \pm 0.0063}$	$\mathbf{0.6366 \pm 0.0099}$	$\mathbf{0.8143 \pm 0.0088}$

The results are presented as *Mean *± * Standard Deviation*. The best results are highlighted in **bold**. DiST consistently achieves superior performance in preserving domain interfaces and preventing over-smoothing.

In terms of global spatial clustering, DiST achieves a remarkable ARI of **0.5900** on the high-resolution **Stereo_AB** dataset, surpassing the second-best method (STAGATE) by a substantial margin of *∼ ***15%**. Similarly, on the **ST_Hip** dataset, which features distinct laminar structures, DiST attains an ARI of **0.6186**, effectively refining the anatomical boundaries compared with SpaGCN (0.5614). Even on the challenging **SeqFISH** embryo dataset, where tissue architecture is complex and dynamic, DiST maintains superior performance (**0.4516**), whereas baselines like STAGATE struggle (0.2760).

Furthermore, the boundary evaluation explicitly highlights DiST’s unique advantage in preventing over-smoothing and preserving sharp domain interfaces. On the **Stereo_DPI** dataset, DiST reaches a striking BF1 of **0.8214**, substantially outperforming the runner-up GraphST (0.6982). This boundary precision is equally pronounced on the **SeqFISH** dataset, where DiST’s BF1 score of **0.7988** easily exceeds SpaGCN (0.7130) and SEDR (0.7060). These improvements confirm that DiST successfully delineates crisp spatial domains even in dynamic or fine-grained architectures.

Finally, the low standard deviations observed across both metrics and all experiments further confirm the stability of our tensor-based SSL framework. **Qualitative visualization.** To intuitively visualize the structural recovery capability of DiST, we present the spatial domain identification results on the Human Breast Cancer (**10X_BC**) dataset in Fig. 3. **Due to space constraints, additional visualizations for the remaining datasets are available in the Supplementary Material** As illustrated in Fig. 3a, the manual annotation reveals a complex heterogeneity of tumor subtypes (e.g. IDC, DCIS/LCIS) and immune environments. In Fig. 3c, conventional methods struggle to capture this complexity: SEDR produces highly fragmented and noisy clusters (ARI=0.4099), while STAGATE and DeepST tend to over-smooth the boundaries between the tumor edge and healthy tissue. The heterophilic GNN baselines also show limited boundary delineation (Geom-GCN ARI = 0.5953; BernNet ARI = 0.4819). In contrast, DiST generates coherent spatial domains with sharp boundaries that align closely with the manual annotations, achieving the highest ARI of **0.6160**. We note that the ARI scores annotated in Fig. 3 are from the representative run used for the spatial-domain visualization, whereas Tables 1 and 2 report the mean *± * standard deviation over 10 random seeds. This qualitative superiority is further corroborated by the UMAP visualization in Fig. 3d. DiST exhibits a clear separation of distinct spatial domains with compact intra-cluster structures, whereas the latent embeddings of baselines remain entangled and less discriminative.

**Figure 3:**
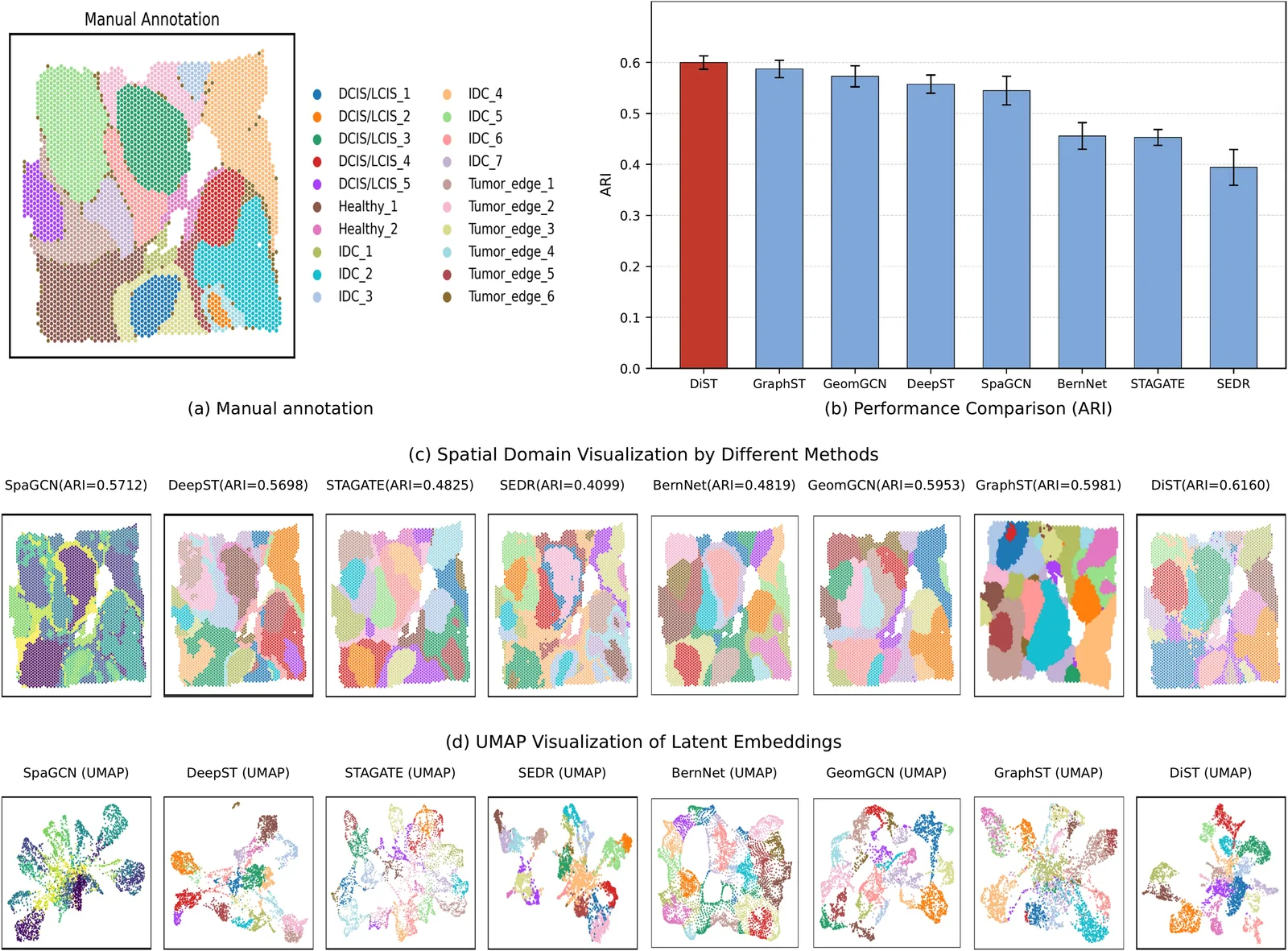
Visualization of spatial domain identification on the 10X_BC dataset. (a) Ground truth annotation. (b) Bar plot of ARI scores comparing DiST against seven representative baselines, including the heterophilic GNN methods Geom-GCN and BernNet. (c) Spatial domains generated by each method, with specific ARI scores listed below. DiST effectively delineates fine-grained tumor boundaries compared with baselines. (d) UMAP visualization of the learned latent embeddings, showing the superior cluster separation achieved by DiST. The ARI scores annotated in (b) and (c) correspond to the representative run used for this visualization, whereas Tables 1 and 2 report the mean *± * standard deviation over ten random seeds.

### Biological interpretability and functional analysis

To validate the capability of DiST in dissecting cancer tissue heterogeneity at a refined molecular level, we conducted a comprehensive case study on the Human Breast Cancer dataset. We specifically examined the expression patterns of top marker genes across four distinct spatial domains identified by DiST: Cluster 0 (Healthy), Cluster 1 (Ductal Carcinoma In Situ, DCIS/LCIS), Cluster 16 (Invasive Ductal Carcinoma, IDC), and Cluster 10 (Tumor Edge). Beyond quantitative clustering metrics, we further characterized these domains through multisource pathway enrichment, STRING PPI network analysis, and linkage to an independent single-cell reference (Fig. 5).

**Figure 5:**
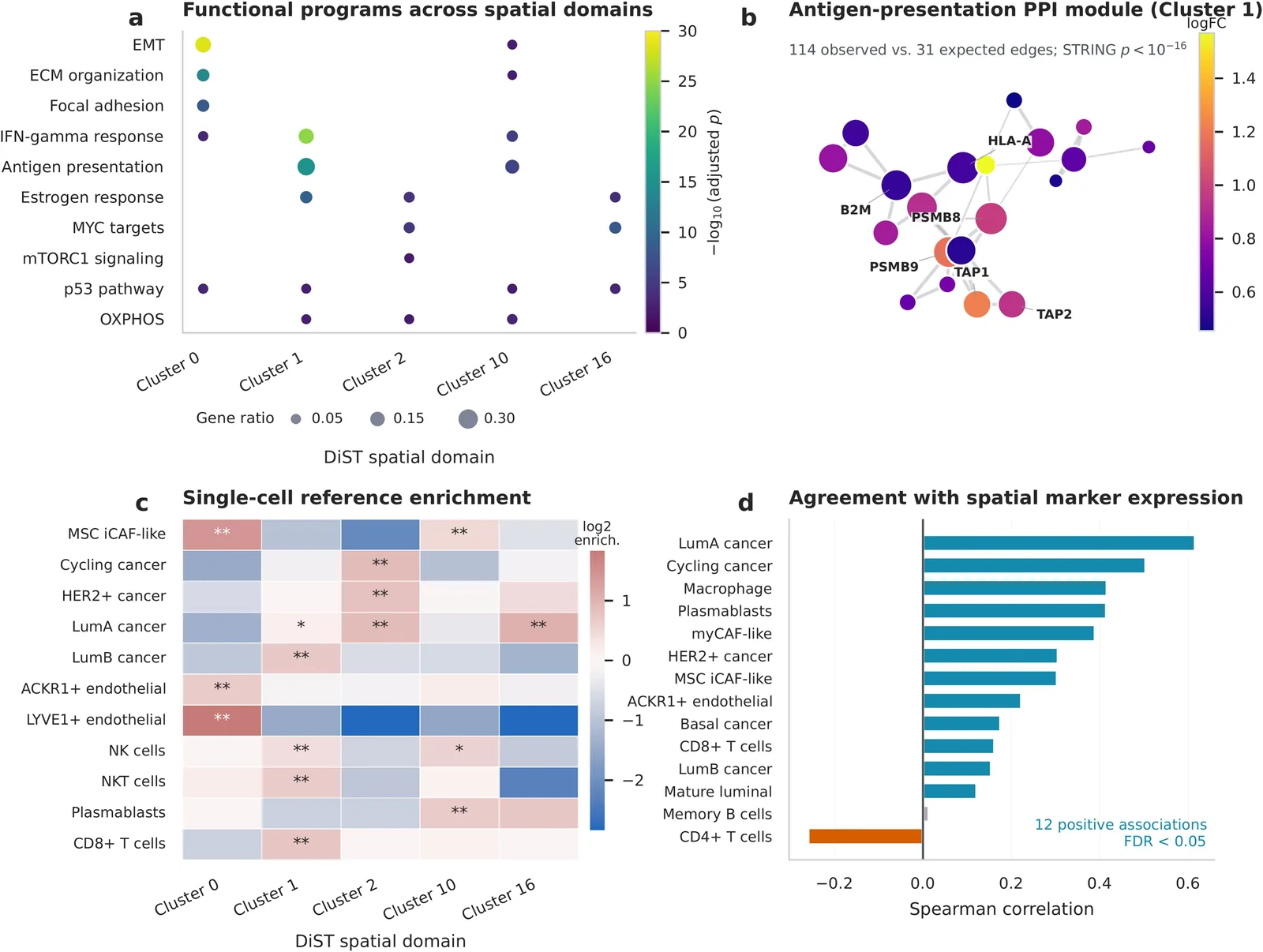
Biological characterization of DiST spatial domains using pathway, PPI, and external single-cell reference analyses. (a) Selected significantly enriched functional programs obtained from KEGG 2021 Human, MSigDB Hallmark 2020, and Reactome 2022 across representative DiST domains (Clusters 0, 1, 2, 10, and 16), analyzed in addition to GO Biological Process. Dot size indicates the gene ratio and color indicates $-\log _{10}$(adjusted *p*-value). (b) STRING PPI network of differentially expressed genes in Cluster 1, highlighting the antigen-processing and presentation module. Node size represents network degree, node color represents differential-expression log fold change, and edge width reflects STRING confidence; the network contains 114 observed versus 31 expected edges (PPI enrichment $p < 10^{-16}$). (c) Enrichment of selected GSE176078 minor cell types across DiST domains, shown as $\log _{2}$ enrichment relative to all spots. Asterisks denote FDR-adjusted significance ($^{*}P<.05$, $^{**}P<.01$, $^{***}P<.001$). (d) Concordance between Tangram-derived cell-type scores and the corresponding spatial marker-module expression. Bars show Spearman correlations; blue and orange denote significant positive and negative associations, respectively, at FDR *< 0.05*, and gray denotes a nonsignificant association.

**Uncovering heterogeneity via marker genes.** As visualized in the expression heatmap (Fig. 4a), DiST reveals significant heterogeneity among these clusters, with each domain exhibiting a unique gene signature. By aligning these signatures with literature, we confirmed the biological accuracy of our partitions.

- **DCIS/LCIS (Cluster 1):** We identified *CPB1* and *IL6ST* as top marker genes. High expression of *CPB1* has been reported as a specific prognostic marker for ductal carcinoma in situ [39], while *IL6ST* plays a potential role in mediating endocrine resistance via the ER signaling pathway [40].
- **Tumor Edge (Cluster 10):** This transition zone is characterized by the co-expression of *KRT19* and *APOE*. *KRT19* has been found to directly interact with the *β *-catenin/RAC1 complex to regulate the NOTCH signaling pathway [41], indicating an active signaling environment at the boundary. Concurrently, the detection of *APOE* aligns with studies linking Apolipoprotein E polymorphisms and serum lipoproteins to breast cancer risk [42], suggesting a complex metabolic landscape in the tumor microenvironment.
- **IDC (Cluster 16):** In the invasive region, DiST detected elevated expression of *RPLP1* and *SH3BGRL*. *RPLP1* is known for its aberrant expression in breast tumors [43], and *SH3BGRL* has been implicated in driving autophagy-mediated chemoresistance and promoting stability in breast cancers [44]. Additionally, the upregulation of *COL2A1* suggests extracellular matrix remodeling typical of invasive processes [45].

**Figure 4:**
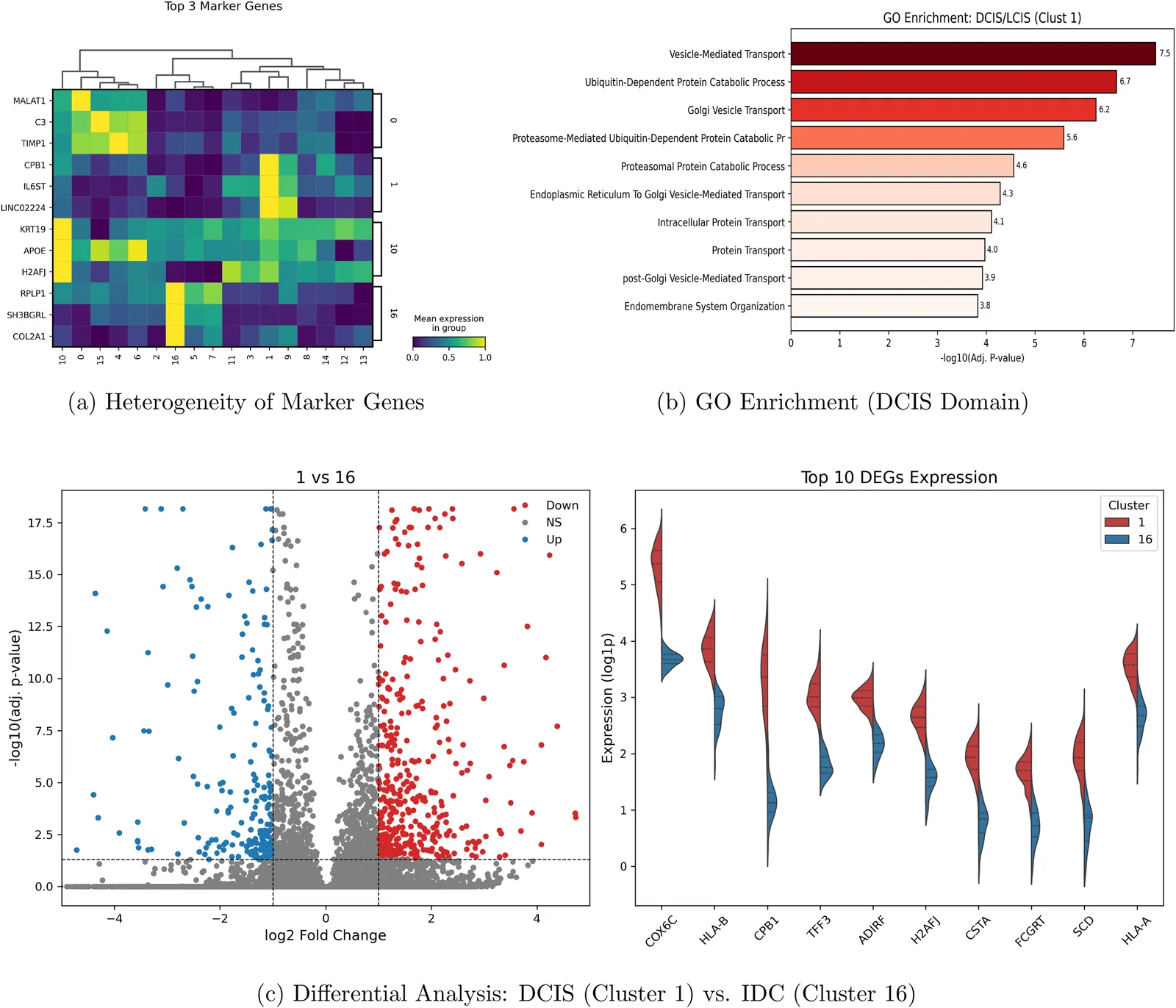
Biological interpretability and functional validation on the Human Breast Cancer dataset. (a) Heatmap visualizing the expression levels of top 3 marker genes across four distinct spatial domains: Healthy (0), DCIS/LCIS (1), Tumor Edge (10), and Invasive Ductal Carcinoma (IDC, 16), revealing clear molecular heterogeneity. (b) GO enrichment analysis for the DCIS domain (Cluster 1), highlighting active pathways related to vesicle-mediated transport. (c) Pairwise differential expression analysis between the non-invasive DCIS domain and the invasive IDC domain. The volcano plot (left) and violin plots (right) illustrate significant transcriptional changes driving the progression of malignancy.

**Differential expression and pathway analysis.** To further investigate the molecular mechanisms driving the transition from non-invasive to invasive states, we performed a pairwise differential expression analysis between the DCIS domain (Cluster 1) and the IDC domain (Cluster 16). As shown in the volcano plot (Fig. 4c), we detected significant transcriptional differences, identifying distinct DEGs that mark the progression of malignancy. The violin plots (Fig. 4c, right) further illustrate the sharp contrast in expression levels of top DEGs between these two clusters.

Furthermore, to elucidate the functional characteristics of the DCIS domain (Cluster 1), we conducted GO enrichment analysis. As presented in Fig. 4b, this region is highly enriched in terms related to **“Vesicle-Mediated Transport”** and **”Protein Catabolic Process.”** The prominence of vesicle transport suggests an active secretory phenotype, likely involving the release of extracellular vesicles or signaling factors to modulate the local microenvironment prior to invasion. Simultaneously, the enrichment of protein catabolic processes indicates high protein turnover, which is essential for maintaining cellular homeostasis under the metabolic stress of confinement within the ducts. This functional profile captures the biological behavior of carcinoma cells that are spatially constrained yet metabolically hyperactive.

**Pathway enrichment across spatial domains.** Beyond GO Biological Process enrichment, we performed pathway-level enrichment analysis of the DEGs of each DiST domain using three complementary pathway collections: KEGG 2021 Human, MSigDB Hallmark 2020, and Reactome 2022. As shown in Fig. 5a, the DiST domains exhibit distinct functional programs: Cluster 0 is characterized by Hallmark epithelial-mesenchymal transition, Reactome extracellular-matrix organization, and KEGG focal-adhesion programs; Cluster 1 is dominated by Hallmark interferon-gamma response and KEGG antigen processing and presentation; Clusters 2 and 16 are associated with Hallmark estrogen-response and MYC-target programs; and additional programs such as mTORC1 signaling, the p53 pathway, and oxidative phosphorylation are differentially activated across the domains. These results indicate that each DiST domain corresponds to a coherent, pathway-level biological program rather than a purely statistical partition.

**PPI network analysis.** To examine the interaction structure underlying the domain-specific DEGs, we performed STRING PPI enrichment analysis. In the five representative domains, the DEGs form significantly connected PPI networks, with observed/expected edge counts of 107/30 for Cluster 0 (3.57-fold, PPI enrichment $P < 10^{-16}$), 114/31 for Cluster 1 (3.68-fold, $P < 10^{-16}$), 64/42 for Cluster 2 (1.52-fold, *P = 0.0012*), 250/98 for Cluster 10 (2.55-fold, $P < 10^{-16}$), and 1586/356 for Cluster 16 (4.46-fold, $P < 10^{-16}$). In particular, as visualized in Fig. 5b, the Cluster 1 network contains a coherent antigen-presentation module centered on *HLA-A*, *HLA-B*, *B2M*, *TAP1*, *TAP2*, *PSMB8*, and *PSMB9*, providing network-level support for the pathway-enrichment result.

**Linking spatial domains to single-cell references.** To link the DiST clustering results with single-cell references, we mapped the 10X_BC spatial data to 29 cell types of the independent breast-cancer single-cell RNA-seq reference GSE176078 [38] using Tangram. Because a spatial domain can contain multiple cell populations, we interpret the results as domain-level cellular compositions and microenvironments rather than assigning a single pure cell type to each domain. The inferred compositions differ across DiST domains significantly more than expected under permutation, at both the major-cell-class level ($R^{2} = 0.2305$, permutation $P < 5 \times 10^{-4}$) and the minor-cell-type level ($R^{2} = 0.0917$, permutation $P < 5 \times 10^{-4}$), yielding 12 significant major-class and 29 significant minor-type enrichments (Fig. 5c). Representative associations include iCAF-like and vascular populations in Cluster 0, CD8 T, LumB cancer, macrophage, and NK populations in Cluster 1, LumA, cycling, and HER2+ cancer populations in Cluster 2, and plasmablast, NK, CAF, and macrophage populations in Cluster 10. As an independent validation, we compared each mapped cell-type score with the corresponding spatial marker-module expression: 12 cell types show significant positive concordance at FDR *< 0.05* (Fig. 5d), including LumA cancer (Spearman *ρ = 0.616*), cycling cancer (*ρ = 0.504*), macrophages (*ρ = 0.416*), plasmablasts (*ρ = 0.415*), and myCAF-like cells (*ρ = 0.389*).

**Relating tensor components to biological functions.** These analyses also clarify the biological meaning of the tensor components recovered by DiST. The low-rank component $\boldsymbol{\mathcal{H}}_{i}$ retains the expression structure that is coherent across semantically aligned ego-networks; accordingly, the spatial domains derived from it exhibit pathway-coherent programs, densely connected PPI modules, and consistent single-cell reference compositions, as validated above. In contrast, the sparse component $\boldsymbol{\mathcal{S}}_{i}$ captures the sparse, domain-incoherent fluctuations, i.e. the technical dropouts and sporadic artifacts that do not correspond to reproducible biological function. The functional interpretability of the low-rank domains thus provides independent evidence that the decomposition separates biological signal from technical noise rather than merely compressing the data.

In summary, DiST not only identifies spatial domains that align with manual annotations but also recovers biologically meaningful molecular signatures. The consistency between our findings and established cancer literature demonstrates that DiST effectively disentangles authentic biological signals from noise, providing valuable insights for downstream clinical analysis.

### Ablation study

To strictly isolate and evaluate the contribution of the proposed semantic alignment module, we conduct an ablation study by comparing the full DiST framework with a degraded variant that skips the semantic alignment step. Specifically, instead of globally retrieving semantically similar structures, this variant constructs the 3D tensor using spatially adjacent ego-networks only. We denote this baseline as **DiST-Spatial**. Consistent with the implementation, its anchor list is the target spot’s 1-hop neighborhood returned from the spatial graph encoded by edge_index; its size may therefore vary across targets. It is not a coordinate-based Euclidean top-*K* retrieval. This setting mirrors the local aggregation paradigm of traditional SRT methods.

As reported in Table 3, the full DiST model (semantic alignment) substantially outperforms the DiST-Spatial variant across all five datasets. The performance gap is particularly striking on the 10X_BC dataset, where the ARI drops from 0.5998 to 0.5065 when semantic alignment is removed.

**Table 3:** Component-wise ablation study.

**Metric**	**Variant**	**SeqFISH**	**Stereo_AB**	**Stereo_DPI**	**10X_BC**	**ST_Hip**
ARI	Spatially Adjacent (DiST-Spatial)	0.4258 *± * 0.0133	0.5736 *± * 0.0164	0.3866 *± * 0.0087	0.5065 *± * 0.0407	0.5834 *± * 0.0293
	Matrix-Based (Matrix-DiST)	0.3420 *± * 0.0181	0.4098 *± * 0.0184	0.3249 *± * 0.0191	0.5683 *± * 0.0451	0.5440 *± * 0.0146
	Semantic Alignment (DiST)	**0.4516 *± * 0.0071**	**0.5900 *± * 0.0069**	**0.4288 *± * 0.0087**	**0.5998 *± * 0.0131**	**0.6186 *± * 0.0123**
Boundary F1	Spatially Adjacent (DiST-Spatial)	0.7804 *± * 0.0113	0.6921 *± * 0.0282	0.7510 *± * 0.0155	0.5874 *± * 0.0167	0.7718 *± * 0.0197
	Matrix-Based (Matrix-DiST)	0.6018 *± * 0.0078	0.6367 *± * 0.0304	0.6404 *± * 0.0223	0.5574 *± * 0.0156	0.6741 *± * 0.0104
	Semantic Alignment (DiST)	**0.7988 *± * 0.0055**	**0.7287 *± * 0.0061**	**0.8214 *± * 0.0063**	**0.6366 *± * 0.0099**	**0.8143 *± * 0.0088**

Comparison of DiST with two degraded variants: (i) **DiST-Spatial**, which constructs the tensor from spatially adjacent ego-networks without semantic alignment, and (ii) **Matrix-DiST**, which replaces the low-rank and sparse tensor decomposition with matrix robust PCA on the first-mode unfolding (implementation axis 0) of the constructed tensor, while keeping all other components identical. ARI and Boundary F1 are reported across five benchmark datasets. Best results per metric are in **bold**.

This significant degradation confirms our fundamental hypothesis: physical proximity does not strictly guarantee biological similarity. By relying exclusively on spatially adjacent ego-networks, the model is forced to aggregate distinct transcriptomic profiles at tissue boundaries (e.g. mixing healthy and invasive tumor spots), thereby introducing severe structural noise. In contrast, by skipping the rigid spatial constraints and employing semantic alignment, DiST successfully identifies functional dependencies across distant regions, preserving the purity of the constructed low-rank tensor.

**Matrix-based variant (Matrix-DiST).** To empirically evaluate the contribution of the tensor structure, we implement a matrix-based variant, Matrix-DiST, which replaces the low-rank and sparse tensor decomposition with conventional matrix robust PCA applied to the first-mode unfolding (implementation axis 0) of the constructed tensor, i.e. the matrix obtained by stacking all views as rows. All preprocessing, anchor selection, hop numbers, optimization settings, and clustering procedures are kept identical to DiST, so that the comparison isolates the effect of preserving the tensor modes. As reported in Table 3, DiST consistently outperforms Matrix-DiST across all five datasets in both ARI and Boundary F1 (e.g. on the Stereo_DPI dataset, the ARI improves from 0.3249 to 0.4288 and the Boundary F1 from 0.6404 to 0.8214). This controlled comparison, together with the analysis in the Method section, provides empirical evidence that retaining the high-order tensor layout is beneficial under the evaluated settings.

### Optimization behavior of ADMM

To examine the numerical behavior of the ADMM solver independently of the fixed 10-iteration budget used to generate the reported representations, we perform a *post hoc* diagnostic run for 50 iterations on 30 sampled ego-network tensors per dataset. We record the normalized reconstruction residual $r_{t}=\|\boldsymbol{\mathcal{H}}_{i}^{t}+\boldsymbol{\mathcal{S}}_{i}^{t}-\boldsymbol{\mathcal{X}}_{i}\|_{F}/\|\boldsymbol{\mathcal{X}}_{i}\|_{F}$ and the normalized iterate change $q_{t}$, defined as the maximum of the relative changes of $\boldsymbol{\mathcal{H}}_{i}$ and $\boldsymbol{\mathcal{S}}_{i}$. Figure 6 reports the median curves and interquartile ranges across the sampled tensors. Both diagnostics decrease rapidly during the early iterations and continue to decline thereafter. The dashed line at $10^{-5}$ is a visual reference for numerical accuracy, not the stopping rule used by the fixed-budget production pipeline. Because some median iterate-change curves remain above this reference at iteration 50, the figure is interpreted as evidence of stable numerical descent rather than as a formal claim that every tensor has converged to a common tolerance.

**Figure 6:**
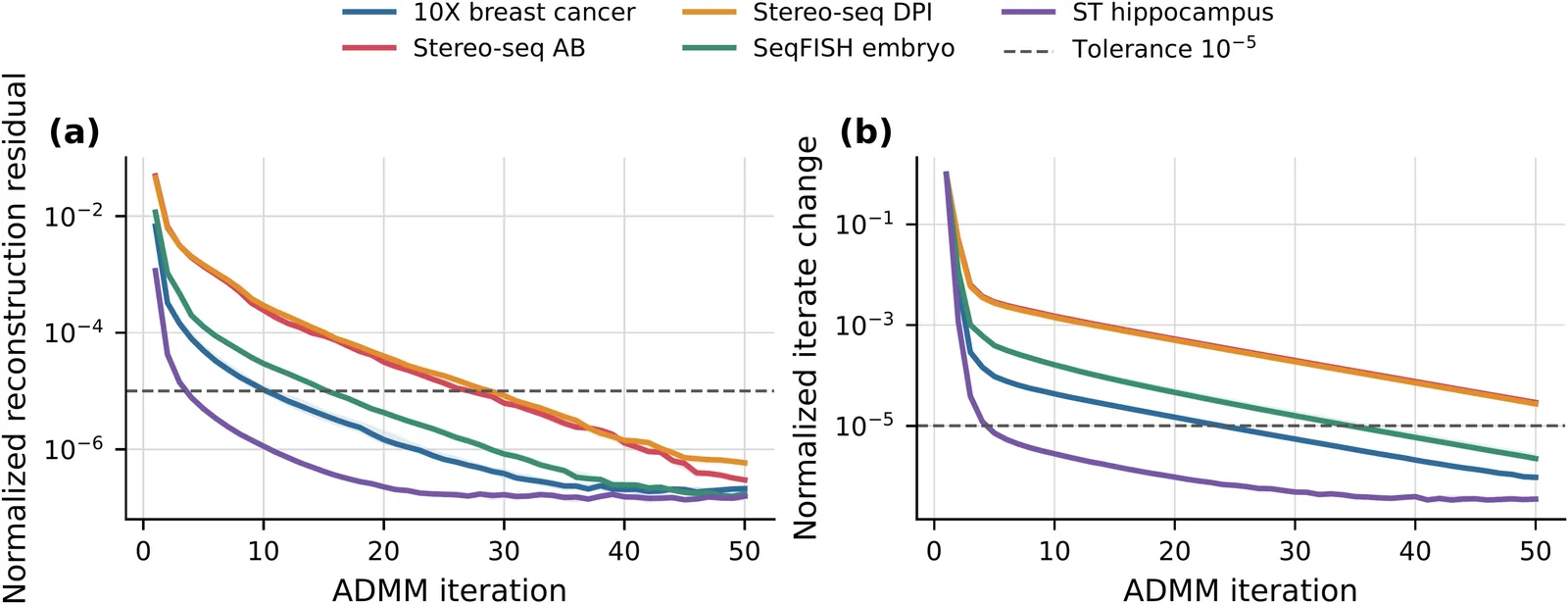
Optimization behavior of the ADMM solver in a 50-iteration *post hoc* diagnostic run. (a) Normalized reconstruction residual and (b) normalized iterate change versus iteration. The solid lines and shaded bands show the medians and interquartile ranges over 30 sampled tensors per dataset. The dashed line at $10^{-5}$ is a numerical reference and is not the stopping rule of the fixed 10-iteration production pipeline.

The sparsity weight *λ * in Equation (4) is set following the dimensional scaling used in TRPCA [30]: $\lambda _{i}=c/\sqrt{\max (3K,D_{i})\,M}$, where *c* is a scaling factor. We evaluate $c\in \{0.8,1.0,1.2,1.4,1.6\}$ in a sensitivity analysis and use *c=1.0*, the standard TRPCA scaling, as the common default for all datasets.

### Robustness to sequencing dropouts

A persistent challenge in SRT is that sequencing dropouts are neither uniform nor random: capture failures tend to be gene-preferential and spatially aggregated, e.g. arising from low-quality tissue patches or expression-dependent capture. To evaluate DiST under such realistic noise, we construct a fully synthetic spatial transcriptomics dataset of 2500 spots *× * 1000 genes organized into five spatial domains, and simulate sequencing noise as a nested heterogeneous capture-thinning process that combines gene-specific and spot-specific capture efficiencies, expression-dependent capture, and spatially correlated low-quality patches. Three noise levels with controlled UMI loss rates of 10%, 20%, and 30% are generated. We compare DiST with SEDR and DeepST on spatial clustering (ARI and Boundary F1) and, as an additional evaluation metric, on gene imputation accuracy, measured by the mean absolute error (MAE) over the masked (lost) entries.

As reported in Table 4, DiST consistently achieves substantially higher ARI and Boundary F1 than both baselines at every dropout level. Even at a 30% UMI loss, DiST retains an ARI of 0.8054 and a Boundary F1 of 0.9913, whereas the ARI of SEDR and DeepST drops to 0.6118 and 0.5541, respectively. For gene imputation, DiST attains the lowest masked MAE at 10% and 20% dropout (0.4643 and 0.4830, versus 0.5462 and 0.5158 for SEDR), and at 30% dropout the methods reach a similar magnitude (DiST 0.5416, SEDR 0.5121, DeepST 0.6281). These results confirm that, in addition to clustering, DiST effectively recovers the expression of entries lost to realistic sequencing dropouts.

**Table 4:** Robustness and gene imputation on the synthetic ST dataset under realistic dropout patterns.

**Method**	**ARI**			**Boundary F1**			**Masked MAE**		
	**10%**	**20%**	**30%**	**10%**	**20%**	**30%**	**10%**	**20%**	**30%**
DiST	$\mathbf{0.8461} \pm {0.0125}$	$\mathbf{0.8340} \pm{0.0120}$	$\mathbf{0.8054} \pm{0.0195}$	$\mathbf{0.9949} \pm{0.0010}$	$\mathbf{0.9942} \pm{0.0011}$	$\mathbf{0.9913} \pm{0.0019}$	$\mathbf{0.4643} \pm{0.0002}$	$\mathbf{0.4830} \pm{0.0002}$	0.5416 *± * 0.0010
SEDR	0.6989 *± * 0.2571	0.6095 *± * 0.2849	0.6118 *± * 0.2822	0.9511 *± * 0.1041	0.9057 *± * 0.1483	0.9025 *± * 0.1526	0.5462 *± * 0.0002	0.5158 *± * 0.0002	$\mathbf{0.5121} \pm{0.0001}$
DeepST	0.5638 *± * 0.0027	0.5591 *± * 0.0040	0.5541 *± * 0.0086	0.8349 *± * 0.0017	0.8356 *± * 0.0025	0.8406 *± * 0.0228	0.4977 *± * 0.0016	0.5448 *± * 0.0024	0.6281 *± * 0.0140

ARI, Boundary F1, and masked MAE of DiST, SEDR, and DeepST under controlled UMI loss rates of 10%, 20%, and 30%. Masked MAE measures the mean absolute error of the predicted expression on the entries lost to dropout. Results are the mean *± * standard deviation over 10 random seeds. Best results per column are in **bold**.

This sustained robustness is consistent with the design of our tensor-based structural learning module. Rather than relying solely on individual gene expression profiles or localized smoothing, which are vulnerable to dropouts, DiST models each constructed tensor as the sum of a low-rank component and an entrywise sparse component. The $\ell _{1}$ penalty encourages dropout-associated deviations to enter the sparse component, while semantic-anchor stacking supplies correlated context from transcriptomically similar spots. The decomposition does not guarantee perfect separation of every structured dropout, but the synthetic results show that the resulting representation remains useful under the evaluated noise patterns.

## Conclusion

To address over-smoothing at tissue boundaries and sequencing dropouts in SRT, we propose **DiST**. This novel self-supervised framework **moves beyond** conventional scalar message passing by leveraging **high-order tensor modeling**. By integrating semantically aligned ego-network tensors with low-rank and sparse decomposition, DiST provides a principled regularization framework for learning tissue representations from noisy data. DiST consistently outperforms the evaluated baselines across five datasets and delineates invasive tumor-associated regions in the breast cancer case study. These results support DiST as a useful framework for analyzing complex tissue architectures in spatial transcriptomics.

**Key Points**

- **Problem identification:** the homophily assumption in existing GNN-based spatial transcriptomics methods causes over-smoothing at tissue boundaries. This heterophily problem was validated through our quantitative analysis.
- **Novel framework:** we propose DiST, a self-supervised learning framework that leverages tensor modeling of local ego-networks. This design captures high-order spatial contexts without blurring local details.
- **Robust decomposition:** a low-rank and sparse tensor model separates a correlated representation component from sparse entrywise deviations, improving robustness to sequencing dropouts in the evaluated datasets.

## Supplementary Material

Supplementary_Material_bbag600

## Data Availability

The spatial transcriptomics datasets analyzed in this study are publicly available from their respective sources: The 10x Visium Human Breast Cancer data (10X_BC) is available from the 10x Genomics dataset page. The ST Mouse Hippocampus data (ST_Hip_1) is accessible via Mendeley Data. The Stereo-seq Axolotl Brain data (Stereo_AB and Stereo_DPI) can be obtained from the STOmics DB. The SeqFISH Mouse Embryo data (SeqFISH_embro_1) is available in the SpatialMouseAtlas2020 GitHub repository . The DiST source code is publicly available at https://github.com/LYZ1222/DIST. It includes preprocessing, spatial graph and tensor construction, ADMM decomposition, clustering, and the experimental configurations (hyperparameters, cluster numbers, and random seeds 2023–2032).
