# Supplementary material for: DiST: disentangling structure via self-supervised tensors for heterophilous spatial transcriptomics
Source: Brief Bioinform. 2026 Oct 30;27(5):bbag600. doi: 10.1093/bib/bbag600 (PMC13720745; doi:10.1093/bib/bbag600)
Supplement: Supplementary file 1 [file supplementary_material_bbag600.pdf]

Supplementary Material

DiST: Disentangling Structure via Self-Supervised Tensors for Heterophilous Spatial Transcriptomics

Supplementary Note 1 Additional Benchmark Visualizations

Due to space constraints in the main text, we provide the complete spatial domain identification results for the remaining four datasets in this section. Detailed analyses and visualizations for the Axolotl Brain (Control & DPI), Mouse Hippocampus, and Mouse Embryo datasets are presented below.

Supplementary Note 1.1 Axolotl Brain (Control)

The visualization results for the Axolotl Brain (Control) dataset are shown in Figure S1. As observed in Figure S1(b), DiST achieves the highest clustering accuracy with a mean ARI of **0.5900 ± 0.0069** (best run 0.6018), outperforming the runner-up STAGATE (mean ARI 0.5145 ± 0.0224) and other baselines.

Visually, Figure S1(c) demonstrates that baselines such as SpaGCN and DeepST suffer from noise. In contrast, DiST effectively eliminates noise while preserving sharp boundaries, yielding spatial domains that closely match the manual annotations (Figure S1(a)). Furthermore, the UMAP visualization in Figure S1(d) confirms that the latent embeddings learned by DiST are more compact and distinct.

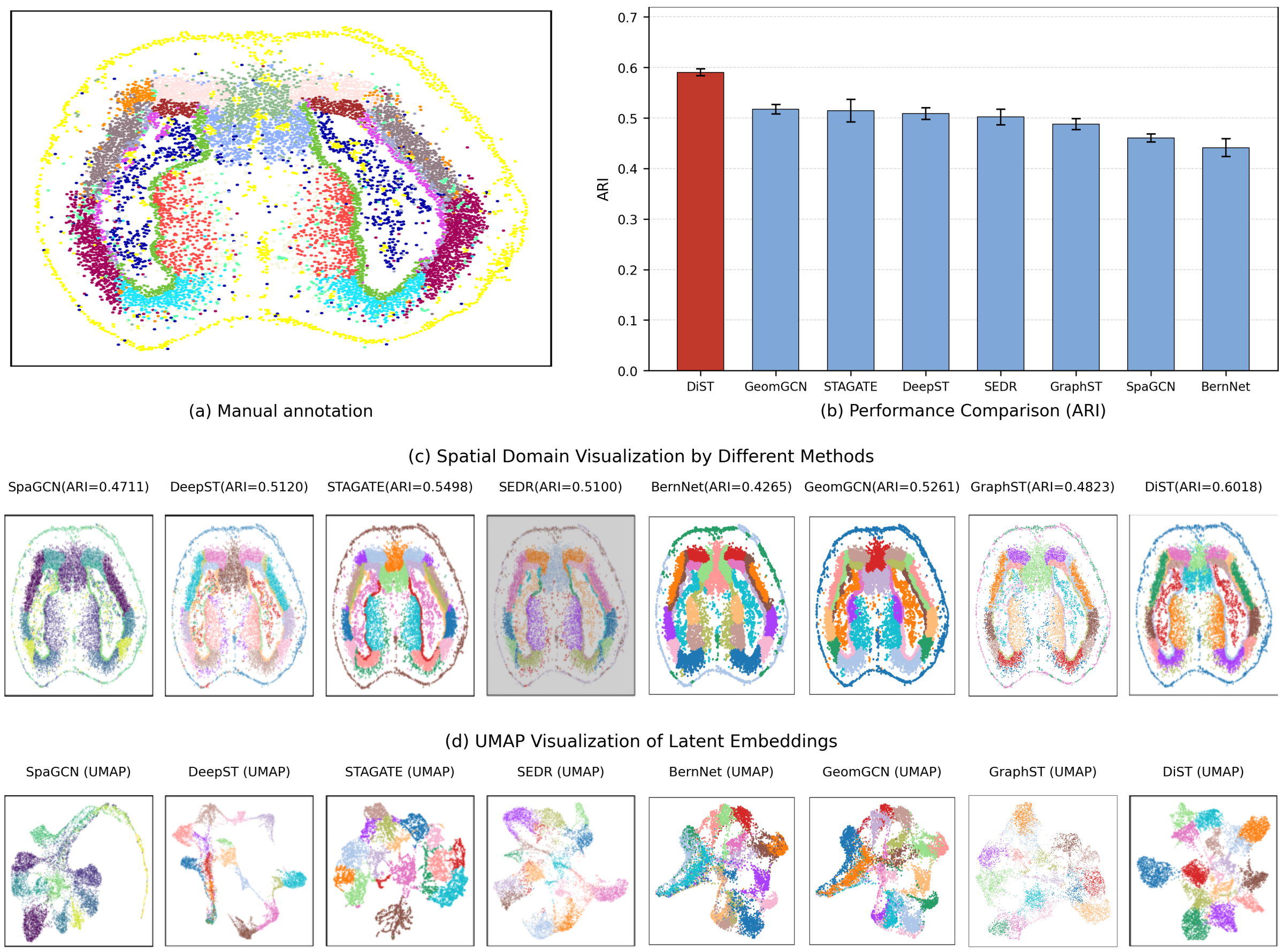

Figure S1. Comparison of spatial domain identification on the Stereo-seq Axolotl Brain (Control) dataset. (a) Manual annotation as ground truth. (b) Bar plot comparing ARI scores (Mean ± Std) of DiST against seven baseline methods, including SRT-specific methods (SpaGCN, DeepST, STAGATE, SEDR, and GraphST) and heterophilic GNNs (BernNet and Geom-GCN). (c) Spatial domains identified by various models, with specific ARI scores listed below. (d) UMAP visualizations of the latent representations.

Supplementary Note 1.2 Axolotl Brain (2 Days Post-Injury)

The visualization results for the Axolotl Brain (2 Days Post-Injury) dataset are detailed in Figure S2.

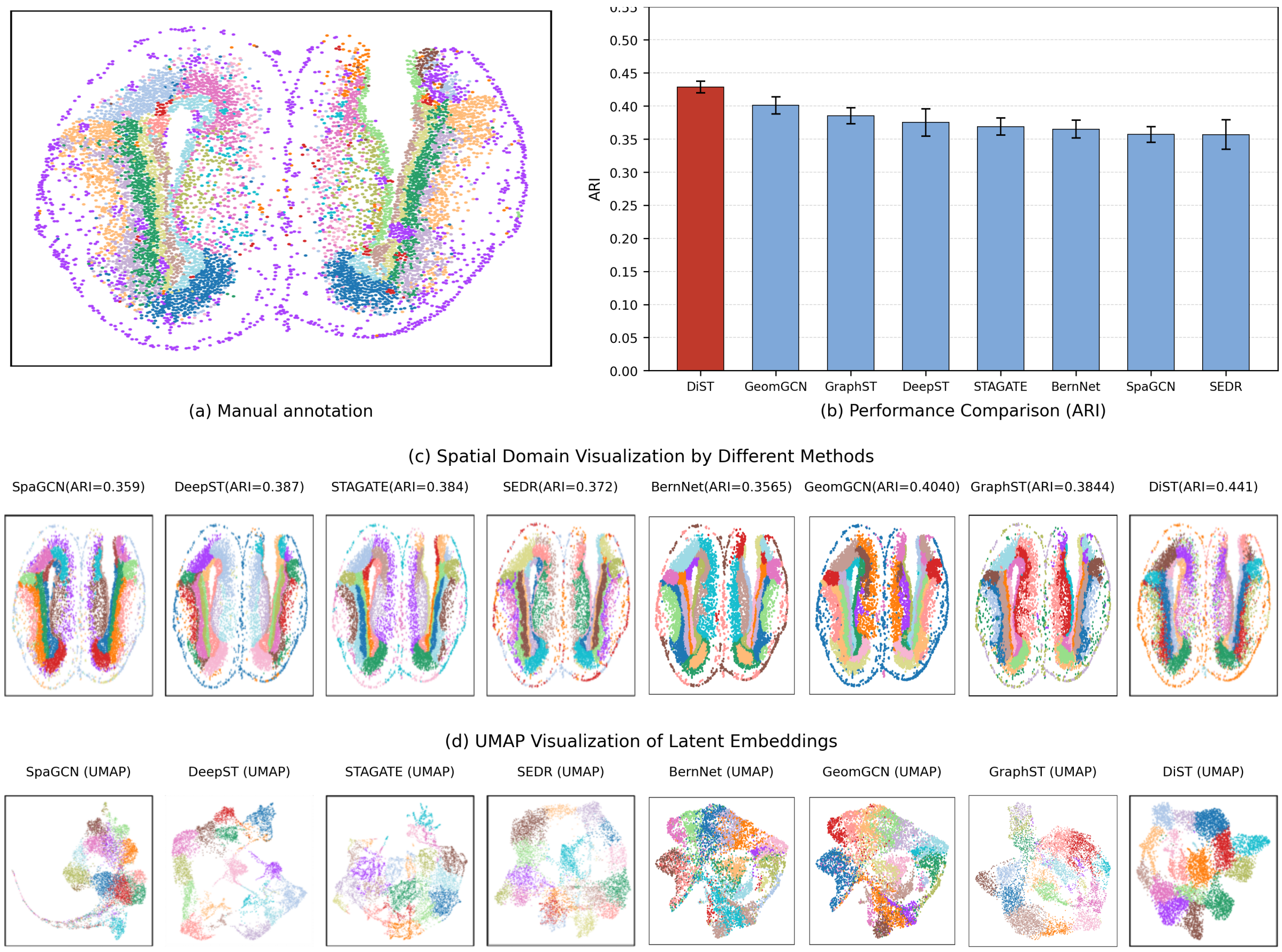

Figure S2. Comparison of spatial domain identification on the Axolotl Brain (2 Days Post-Injury) dataset. (a) Manual annotation. (b) Bar plot comparing ARI scores (Mean ± Std) of DiST against seven baseline methods, including SRT-specific methods (SpaGCN, DeepST, STAGATE, SEDR, and GraphST) and heterophilic GNNs (BernNet and Geom-GCN). (c) Visualization of spatial domains identified by different methods, with specific ARI scores listed below. (d) UMAP projection of the learned embeddings.

Supplementary Note 1.3 Mouse Hippocampus

The Mouse Hippocampus dataset is a classic benchmark for evaluating the ability to recover laminar organization. As illustrated in Figure S3, DiST outperforms all baselines with an impressive mean ARI of **0.6186** $\pm$ **0.0123** (best run 0.635).

The hippocampus structure consists of thin, distinct layers (e.g., Cornu Ammonis and Dentate Gyrus). In the representative run shown in Figure S3(c), GNN-based methods like STAGATE (ARI=0.491) and SEDR (ARI=0.541) tend to over-smooth these thin layers, merging them with adjacent regions due to the homophily assumption. SpaGCN (ARI=0.571), while capturing some structure, exhibits noticeable noise. DiST accurately reconstructs the laminar architecture with sharp boundaries, effectively distinguishing the CA1, CA3, and DG regions. The UMAP visualization in Figure S3(d) further corroborates this, showing a well-structured trajectory that reflects the biological organization of the hippocampus.

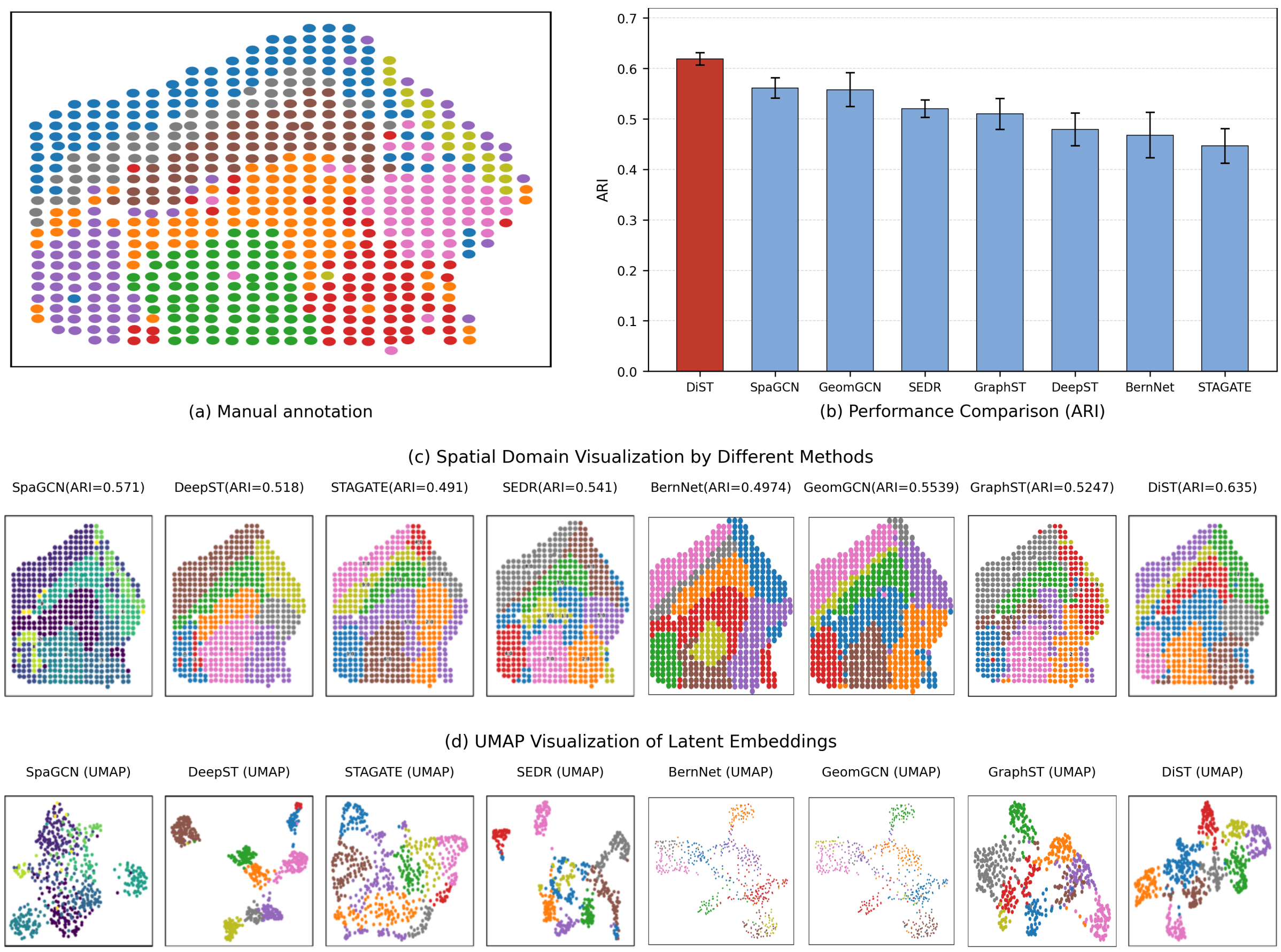

Figure S3. Comparison of spatial domain identification on the Mouse Hippocampus dataset. (a) Manual annotation showing the laminar structure. (b) Bar plot comparing ARI scores (Mean $\pm$ Std) of DiST against seven baseline methods, including SRT-specific methods (SpaGCN, DeepST, STAGATE, SEDR, and GraphST) and heterophilic GNNs (BernNet and Geom-GCN). (c) Visualization of identified domains, with specific ARI scores listed below. (d) UMAP visualization of latent embeddings.

Supplementary Note 1.4 Mouse Embryo (seqFISH)

Finally, we evaluate DiST on the Mouse Embryo dataset (Figure S4), which represents a complex system during early organogenesis with intricate spatial patterns. DiST achieves a mean ARI of **0.4516 ± 0.0071** (best run 0.466), consistently surpassing the baselines. In the representative run shown in Figure S4(c), DeepST and SpaGCN obtain ARI values of 0.4209 and 0.4124, respectively.

The advantage of DiST is particularly evident in identifying small, biologically distinct regions that are often missed by other methods. As seen in Figure S4(c), STAGATE performs poorly (ARI=0.2890), failing to capture the structural diversity of the developing embryo. This failure is likely due to the over-smoothing of local details in such a high-heterogeneity environment. In contrast, DiST preserves these fine-grained details, such as the gut tube and neural crest structures, demonstrating its capability to handle complex tissue architectures even with high resolution.

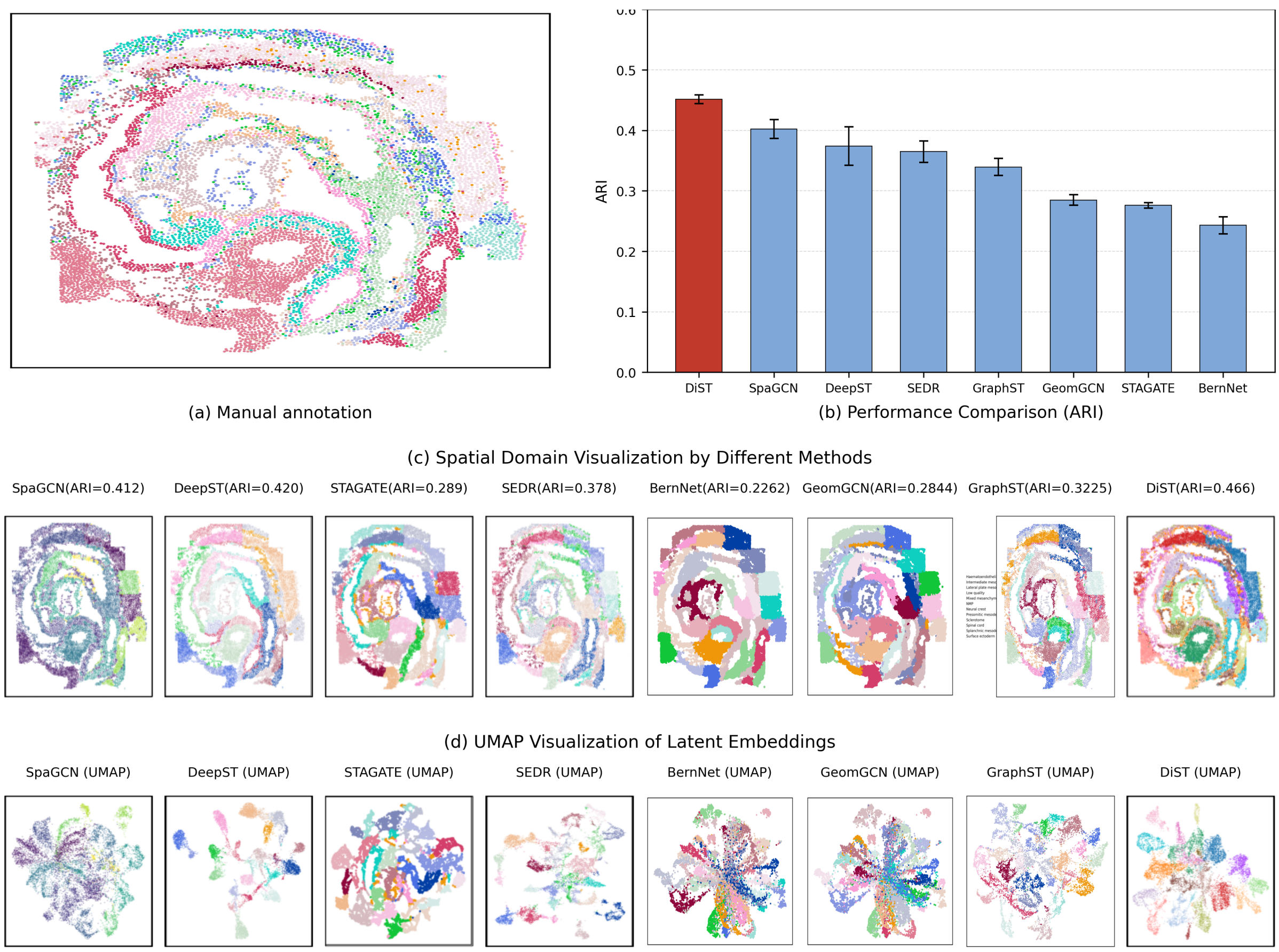

Figure S4. Comparison of spatial domain identification on the Mouse Embryo (seqFISH) dataset. (a) Manual annotation. (b) Bar plot comparing ARI scores (Mean ± Std) of DiST against seven baseline methods, including SRT-specific methods (SpaGCN, DeepST, STAGATE, SEDR, and GraphST) and heterophilic GNNs (BernNet and Geom-GCN). (c) Spatial domain visualization showing DiST's ability to capture fine-grained organ structures, with specific ARI scores listed below. (d) UMAP visualization of latent embeddings.
